# Regulatory mechanisms of programmed cell death in hyperuricaemia and their disease relevance

**DOI:** 10.3389/fcell.2026.1934197

**Published:** 2026-09-30

**Authors:** Heng Sai, Haojun Shen, Jing Fu, Wenhui Liu

**Affiliations:** Hetao College, Bayannur, Inner Mongolia, China

**Keywords:** ferroptosis, hyperuricaemia, NLRP3 inflammasome, programmed cell death, pyroptosis

## Abstract

Hyperuricaemia (HUA) is a metabolic disorder that can not only predispose patients to gout but also be associated with renal, cardiovascular, and metabolic diseases. Recent evidence has designated programmed cell death (PCD) as a central event that drives tissue injury and inflammation in HUA. Raised soluble urate and exposure to monosodium urate (MSU) crystals activate separate but shared stress and injury pathways, including apoptosis, pyroptosis, ferroptosis, and necroptosis in concert with dysregulated autophagy. These processes converge through oxidative stress, mitochondrial dysfunction and signalling along the inflammasome–immune axis to influence disease progression. Among these cell death modalities, the NOD-like receptor family pyrin domain containing 3 (NLRP3) inflammasome–mediated pyroptosis is a crucial amplifier of urate-induced inflammation and concomitant cellular injury; ferroptosis also contributes to irreversible organ damage along with impaired autophagy. More importantly, extensive crosstalk among these pathways collectively shapes disease trajectory and outcomes. This Review summarises the main regulatory mechanisms that promote PCD during HUA, with a focus on renal injury, cardiovascular damage and metabolic dysregulation. Distinct from pathway-specific summaries, this Review integrates apoptosis, pyroptosis, ferroptosis and necroptosis together with autophagy-related dysfunction into a unified disease-relevance framework linking urate-associated stress, pathway crosstalk and organ-specific injury in HUA. Current limitations and novel therapeutic targets are also discussed to inform basic mechanistic studies together with potential clinical strategies.

## Graphical Abstract

**Figure:**
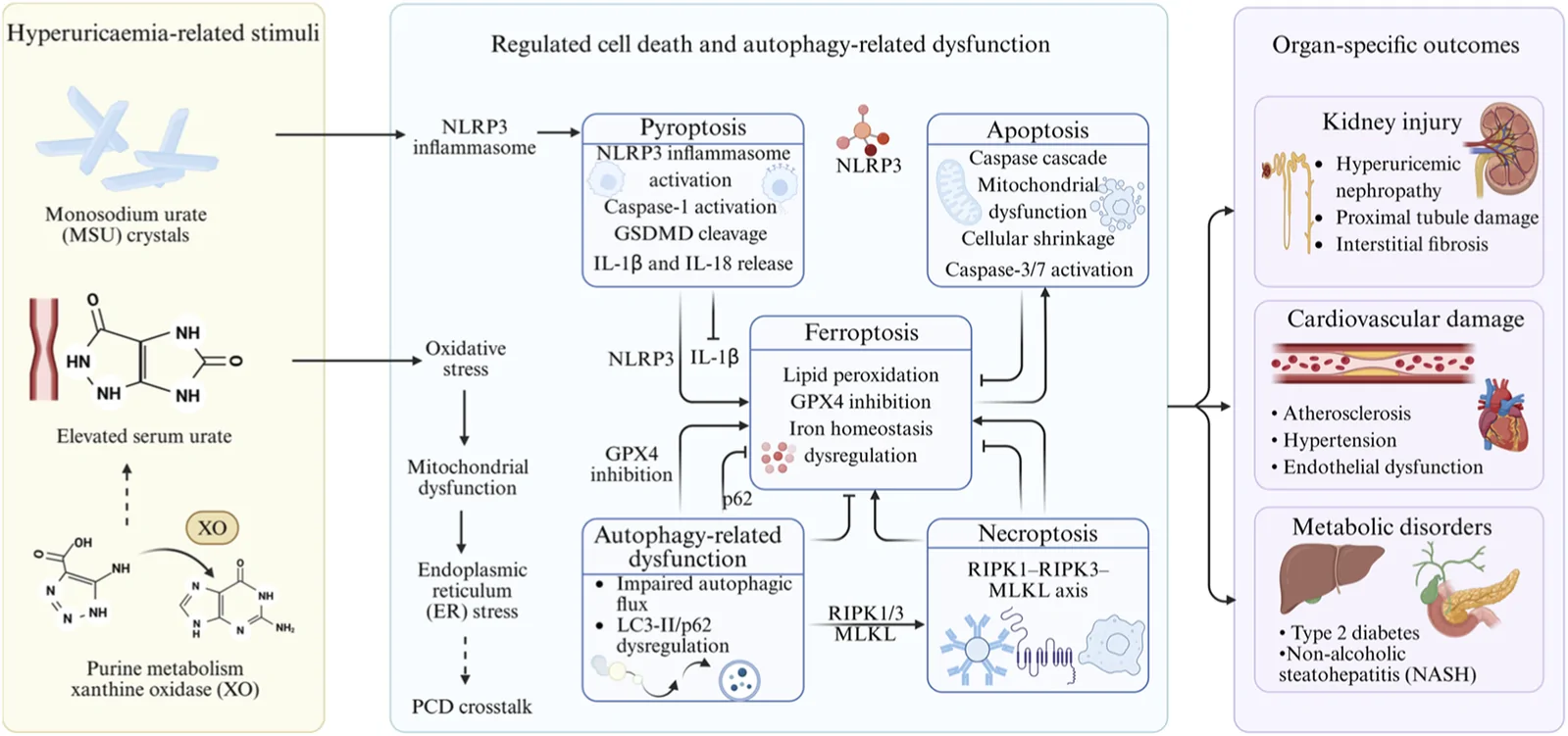

## 1 Introduction

HUA is defined as the persistent elevation of serum urate above a clinically determined threshold; a serum urate concentration of around 6.8 mg/dL (404 μmol/L) approximately corresponds to the solubility limit of urate in human plasma, although diagnostic thresholds may vary by guideline, sex and clinical context (Brikman et al., 2025; Liu et al., 2024). The most common causes of HUA are excessive urate production, diminished renal or intestinal urate excretion, or a combination of both. During the last decades, the prevalence has been growing steadily worldwide due to dietary preferences, sedentary lifestyle and population ageing with the incidence increasing across younger age groups. HUA has become a prominent public health problem worldwide. Urate, the final metabolite during purine metabolism, has antioxidant properties at physiological levels; high urate concentrations can promote oxidative stress in vascular smooth muscle cells (Dogru et al., 2024). Emerging evidence suggests that excessive urate accumulation in the kidney can trigger renal injury, inflammation and fibrosis, thereby promoting the progression of hyperuricaemic nephropathy (HN) (Wang et al., 2024). In view of the increasing mechanistic insight, HUA is not merely seen as a metabolic derangement but rather as a systemic disorder resulting in inflammatory, immune and cellular injury responses. Therefore, its pathogenic mechanisms and relation to diseases are one of the hot topics in current research.

Mechanistically, high soluble urate would need to be differentiated from deposited MSU crystals. On the other hand, soluble urate is dominantly linked to intracellular oxidative stress, mitochondrial dysfunction and metabolic dysregulation, while MSU crystal deposition and phagocytosis can lead to lysosomal rupture, ionic imbalance and vigorous inflammasome activation. While these mechanisms likely converge on shared inflammatory and cell death pathways, in each experimental or disease context, the initiating urate species should be defined.

PCD is an evolutionarily conserved cellular process that participates in the maintenance of tissue homeostasis and includes regulated cell death modalities including apoptosis, pyroptosis, ferroptosis and necroptosis. In stark contrast, autophagy is largely a homeostatic lysosomal degradation pathway and not a mode of cell death. Thus, protective autophagy and impaired autophagic flux are distinct from autophagy-dependent cell death which should only be properly termed as such when the autophagic machinery causally contributes to cell demise. The dysfunction of these pathways contributes to tissue damage and diseases, while coordinating cellular turnover, inflammatory signalling and immune response. In hyperuricaemic environments, regulated cell death pathways such as apoptosis interact closely with dysregulated autophagy, mitochondrial stress signalling and inflammasome activation (Hao et al., 2024). MSU crystal-induced lytic cell death is associated with NLRP3 inflammasome activation, caspase-1 activation and IL-1β release, thereby amplifying inflammation (Pan et al., 2021). Moreover, excess urate accentuates early and late apoptosis in renal proximal tubular epithelial cells (PTECs), which aggravates tubular injury mediated by mitochondrial apoptotic pathways as well as NLRP3 inflammasome activation with gasdermin D (GSDMD)-dependent pyroptosis (Wang et al., 2021). Similarly, compromised autophagy is expected to contribute to urate-associated metabolic dysregulation and inflammatory control. Overall, PCD serves as a central mechanistic bridge linking HUA with organ injury.

Different types of PCD in HUA-dependent diseases run independently and dependently via extensive crosstalk. Caspase cascades, mediators of apoptosis, are a form of regulated cell loss contributing significantly to renal and vascular endothelial injury. Autophagy clears damaged organelles to maintain cellular homeostasis, while its inhibition worsens inflammation and metabolic imbalance. Pyroptosis is a prototypical inflammatory cell death that involves the release of cytokines such as IL-1β and IL-18, which potentiates both local inflammation and systemic responses. However, ferroptosis, an iron-dependent form of regulated cell death characterized by lipid peroxidation and dysregulated iron metabolism, has been proposed as an important mechanism in urate-induced renal injury. Simultaneously, necroptosis and other nonapoptotic pathways were implicated in MSU crystal–induced inflammatory injury (Zhong et al., 2022). Last, accumulating evidence highlights an interplay between the regulation of these death programmes and associated stress pathways. HUA causes endoplasmic reticulum (ER) stress and inflammatory reactions in vascular endothelial cells, accompanied by NLRP3 inflammasome-related signalling. Importantly, the interaction between inflammasome-associated inflammation and ER stress may contribute to urate-induced vascular endothelial dysfunction (ECD) (Ouyang et al., 2022).

Recent advances in the understanding of PCD-related pathways have provided a unique opportunity for intervention against HUA. Pathway- and regulatory-based targets–inhibition of inflammasome activation, modulation of autophagy and suppression of pyroptosis–partially ameliorate urate-induced renal injury (Hu et al., 2022). Nevertheless, the exact regulatory networks and crosstalk between different PCD modalities in HUA are not fully delineated. An in-depth analysis of this context of PCD will sharpen comprehension regarding disease pathogenesis and help to pinpoint targets for drug intervention. Accordingly, this Review provides a summary of the key types of PCD in HUA and their regulatory mechanisms and disease-associated consequences with the intention to guide further research as well as clinical practice.

## 2 Pathophysiological basis of HUA

### 2.1 Uric acid (UA) production and metabolism

Xanthine oxidoreductase (XOR) is the key enzyme in purine catabolism which catalyses hypoxanthine and xanthine to UA (Sekine et al., 2023). UA is the final product of purine catabolism in humans, and since uricase is not present for its further degradation to allantoin, it leads to accumulation within the system. HUA results from excessive urate production and/or decreased urate excretion and is characterized by persistently elevated serum urate levels (Bai et al., 2025). HUA pathogenesis is multifactorial, including disorders of urate metabolism with oxidative stress and inflammatory signalling among multiple PCD pathways. These factors interact to stimulate progressive multi-organ injury, as shown in Figure 1.

**FIGURE 1:**
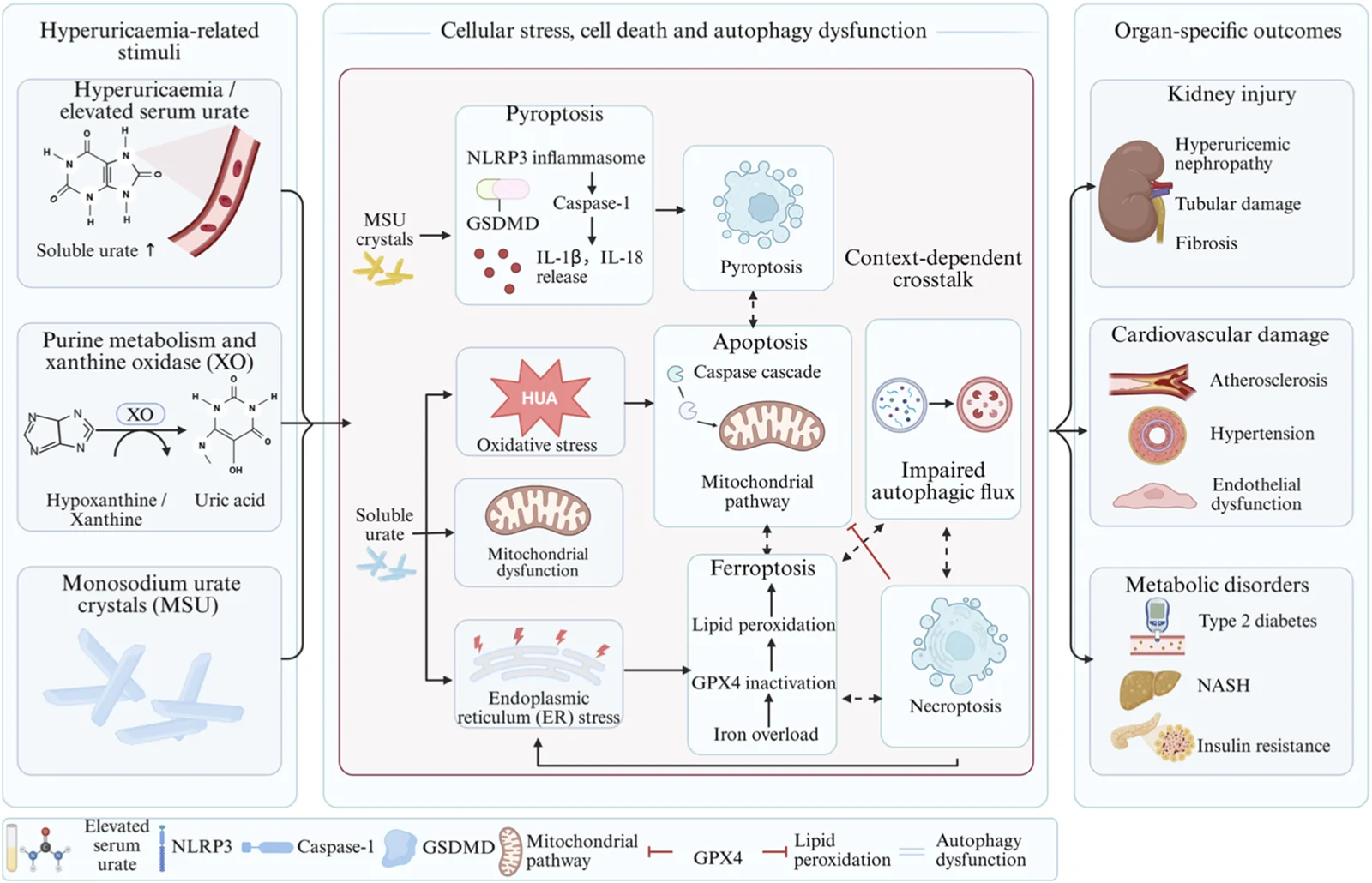
Regulatory mechanisms of PCD and organ injury in HUA: High levels of soluble urate and MSU crystal deposition trigger different but overlapping pathogenic responses in HUA, such as oxidative stress, mitochondrial dysfunction, ER stress, and inflammatory activation. These upstream perturbations fuel increased apoptosis, pyroptosis, ferroptosis and necroptosis, while compromised autophagic flux leads to additional cellular derangement. Crosstalk among these regulated cell death pathways and autophagy dysfunction ultimately elicits exacerbated inflammatory and oxidative injury, thereby participating in the pathogenesis of renal, cardiovascular and metabolic complications associated with HUA.

Altered urate metabolism is more than a metabolic derangement and is tightly associated with oxidative stress as well as inflammatory signalling and apoptosis. Urate metabolic pathways play a central role in acute kidney injury (AKI) and its progression to chronic kidney disease (CKD) (Zhao L. et al., 2025). XOR not only stimulates the production of UA but also produces significant amounts of reactive oxygen species (ROS). Being intermediates in urate synthesis and important sources of ROS, xanthine and hypoxanthine might be protective by allowing their clearance (Arakawa et al., 2020). Excessive ROS disturbs redox homeostasis, and they enhance lipid peroxidation with subsequent DNA damage and pro-inflammatory cytokine release, which aggravates tissue injury. Concurrently, impaired autophagy is now increasingly recognised to be a major player under hyperuricaemic conditions. Defective autophagic flux has effects beyond intracellular homeostasis, as it modulates inflammasome activation linking pyroptosis with inflammation.

Chronic oxidative stress will impair mitochondrial integrity and activity, whereby the triggering of apoptosis in renal tubular epithelial cells mainly occurs due to mitochondrial dysfunction (Yang et al., 2019). Excessive ROS accumulation under hyperuricaemic conditions diminishes mitochondrial membrane potential, attenuates adenosine triphosphate (ATP) production and stimulates cytochrome c efflux to initiate pro-apoptotic signalling cascades. HUA also impairs Na^+^-K^+^-ATPase (NKA)–mediated signal transduction, triggering inflammatory cytokine production and dysfunction of autophagy and mitochondria, resulting in tubular cell injury (Xiao et al., 2019).

The body homeostatically maintains urate levels through regulated production and excretion, with two-thirds of the urate being cleared by the kidneys and one-third in the faeces. The proximal tubule plays a critical role in renal urate handling, where reabsorption and secretion are tightly regulated by various transporters. Of these, urate transporter 1 (URAT1) is the main transporter responsible for tubular reabsorption and a major therapeutic target to achieve lower levels of UA (Li et al., 2019). Several additional transporters are involved in urate transport, including glucose transporter 9 (GLUT9), organic anion transporter 1 (OAT1) and organic anion transporter 3 (OAT3) as well as ATP-binding cassette subfamily G member 2 (ABCG2); dysregulation of their expression or activity prevents the excretion of excess urate and leads to HUA. Together, disordered urate metabolism plays a key role in the pathogenetic mechanisms underlying HUA and its related renal injury, offering mechanistic insights into the analysis of PCD regulation.

### 2.2 Mechanisms underlying HUA

HUA is a metabolic disorder caused by the persistent elevation of serum urate levels due to an increased synthesis and/or decreased renal excretion, which constitutes a crucial pathogenic basis for gout (Matsuo et al., 2014a). UA, the ultimate metabolite of purine metabolism, is closely associated with hepatic insulin resistance (IR), in which HUA-induced autophagy may play a protective role (He F. et al., 2022).

Most of the HUA is due to impaired urate excretion. HUA can be divided into subtypes such as underexcretion, overproduction, mixed forms and extrarenal excretory defects according to renal handling of urate (Qi et al., 2024). Renal urate metabolism involves glomerular filtration and both reabsorption and secretion in the proximal tubule, processes that are closely regulated by a complex array of transporters required to maintain whole body urate homeostasis. URAT1 and GLUT9 are responsible mainly for tubular reabsorption, and OAT1 and OAT3 as well as ABCG2 mediate urate secretion and excretion. ABCG2 is an important high-capacity urate efflux transporter and its deficiency leads to diminished urate clearance, resulting in HUA as well as gout (Matsuo et al., 2014b; Ichida et al., 2012). Polymorphisms in genes linked to urate transport, namely, solute carrier family 2 member 9 (SLC2A9) and solute carrier family 22 member 12 (SLC22A12), are also tightly associated with serum urate levels, a genetic determinant for disease susceptibility and progression.

In addition to hereditary factors and transporter dysfunction, lifestyle factors play an important role in promoting the increasing prevalence of HUA. Urate production is promoted by chronic high-purine intake, alcohol excess and overweight or obesity, as well as by a diet rich in fructose. Interestingly, ATP degradation is promoted by fructose, which, in turn, promotes purine turnover, generating important amounts of urate. IR often accompanies obesity and metabolic syndrome (MetS), and UA is an important risk factor closely associated with hepatic IR (He F. et al., 2022). Although the exact impact of soluble urate on glucose homeostasis is still incompletely defined (Lu et al., 2020), clinical studies have demonstrated a clear association between HUA and diabetes. Recent evidence suggests that urate may contribute to metabolic derangements by acting as a “master” pro-oxidant, inducing oxidative stress, promoting inflammation and mitochondrial dysfunction, thereby exacerbating disturbances in glucose and lipid metabolism and facilitating end-organ injury.

HUA is not merely confined to a metabolic derangement but is intimately associated with chronic inflammation and multi-organ injury. Increased urate levels chronically stimulate inflammation and promote injury of the kidney and cardiovascular systems (Xu Y. X. et al., 2024). MSU crystals deposit and activate the NLRP3 inflammasome to release pro-inflammatory cytokines, including IL-1β, which serves to amplify local inflammation and tissue damage. Persistent NLRP3 inflammasome activation is closely associated with tubulointerstitial fibrosis in HN. Remarkably, bixin can alleviate renal injury and further progression of fibrosis by inducing binding between peroxisome proliferator-activated receptor gamma (PPARγ) and NLRP3 that blocks inflammasome assembly (Cao et al., 2025). The present results integrate dysfunctional urate metabolism, symptomatic genetic predisposition and inflammatory signalling into a cohesive overview of HUA pathogenesis that can inform future work on PCD.

### 2.3 Biological functions of UA

UA, the ultimate end product of human purine metabolism, is produced from xanthine by xanthine oxidase (XO). About two-thirds is removed through the kidneys, with the other third through the intestine. Under normal physiological conditions, urate acts as an antioxidant and is also involved in immune regulation and inflammatory signalling. In contrast, chronic HUA is pathogenic; it initiates and drives disease through mechanisms such as oxidative stress, inflammatory activation and PCD.

UA is an important endogenous antioxidant in human plasma that can scavenge ROS and multiple free radicals, thus preventing lipid peroxidation and oxidative cellular injury (Tian et al., 2021). Urate plays an antioxidant role in maintaining redox homeostasis at physiological concentrations and protects vascular endothelium and neuronal cells. However, its biological function is also context-dependent, and under hyperuricaemic conditions it can act as a pro-inflammatory molecule instead of an antioxidant. Urate is an endogenous damage-associated molecular pattern (DAMP), released from necrotic cells and ischaemic tissues. Acute and chronic gouty inflammation is driven by MSU crystal deposition within joints and periarticular tissues, whereas calcium pyrophosphate dihydrate (CPPD) crystal deposition causes pseudogout (Martinon et al., 2006). Macrophages respond to MSU crystal phagocytosis by secreting cytokines such as IL-1β and tumour necrosis factor-α (TNF-α), which perpetuate the inflammation, hence injuring the surrounding tissues further.

There are strong associations of HUA with vascular injury and metabolic dysfunction. Urate has more recently been regarded as a mechanism of ECD, involving likely central pathways related to inflammation. HUA mechanistically inhibits arginine transport in endothelial cells to induce a suppression of endothelial nitric oxide synthase (eNOS) activity, which ultimately decreases nitric oxide (NO) availability and even worsens ECD. Impaired NO bioavailability impairs vasodilation and serves a pivotal role in initiating cardiovascular disorders such as hypertension and atherosclerosis (Schwartz et al., 2011). Concomitantly, urate activates oxidative stress and inflammatory signalling pathways that perturb lipid metabolism and insulin sensitivity, thus enhancing the development of MetS. Urate is primarily handled in the kidney, an organ that suffers from urate-induced injury. In CKD patients, high serum urate often occurs and prolonged HUA aggravates renal tubular damage, oxidative stress and intrarenal inflammation (Srivastava et al., 2018). PTECs exhibit pronounced expression of pro-inflammatory cytokines and Toll-like receptors in the context of a largely active innate immune response (Milanesi et al., 2019). The accumulation of evidence demonstrates that HUA leads to oxidative stress and mitochondrial dysfunction in the renal cortex, which is involved in the pathogenesis of hypertension associated with urate (Cristóbal-García et al., 2015).

In addition to classical apoptosis, in recent years, there has been an increasing focus on alternative forms of regulated cell death including pyroptosis, necroptosis and ferroptosis, which have all been implicated in urate-mediated renal injury, while dysregulated autophagy can modulate cellular stress responses and susceptibility to these various death pathways. Overproduction of ROS in HUA can engage apoptotic, pyroptotic and ferroptotic signalling pathways; moreover, autophagy dysregulation can further facilitate cellular injury. The collective pleiotropic effects of urate on oxidative stress, inflammatory signalling and PCD form a central pathophysiological axis that underpins disorders associated with HUA.

## 3 Regulated cell death and autophagy-related dysfunction in HUA

### 3.1 Apoptosis

The controlled nature of pathways across signalling networks in tissues and their organs ensures that apoptosis, a ubiquitous form of PCD, is vital for tissue homeostasis. Active apoptotic pathways are observed throughout the kidney, vasculature and metabolically active tissues in HUA, where they likely represent an important mechanism of disease progression. HUA modulates several interconnected cell death programmes via oxidative stress, inflammatory signalling and organelle dysfunction, as shown in Figure 2. UA promotes apoptosis through mitochondrial, death receptor and oxidative stress-dependent pathways. In addition, it reduces phosphorylation of signal transducer and activator of transcription 3 (STAT3) and also inhibits mitochondrial respiration to increase hepatic apoptosis both *in vitro* and *in vivo* (Liu N. et al., 2023).

**FIGURE 2:**
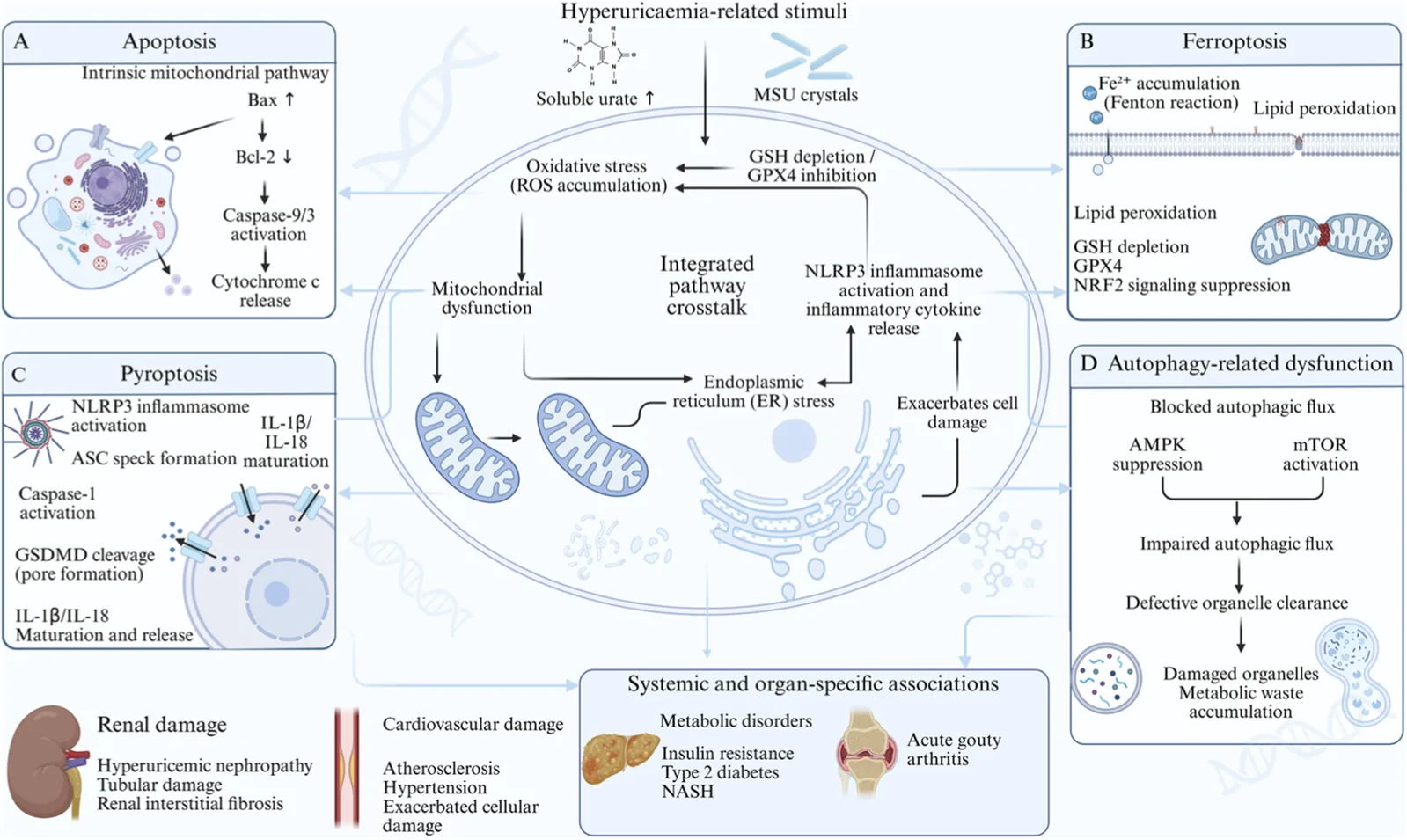
Pathophysiological mechanisms of PCD and autophagy-related dysfunction in HUA: In HUA, soluble urate and MSU crystals activate interlinked stress and inflammatory pathways. **(A)** Mitochondrial dysfunction, Bax/Bcl-2 imbalance and caspase activation are associated with apoptosis. **(B)** Ferroptosis is associated with Fe accumulation, lipid peroxidation and impaired GPX4-dependent antioxidant defence. **(C)** Pyroptosis is driven primarily by NLRP3/caspase-1/GSDMD signalling and inflammatory mediator release. **(D)** Autophagy-related dysfunction is associated with the AMPK/mTOR signalling pathway and may also result from impaired autophagic flux.

Mitochondrial dysfunction is a key mechanism of urate-induced apoptosis, and various experimental interventions reduce tissue injury by modifying mitochondria-mediated intrinsic apoptotic signalling. At the heart of this process are loss of mitochondrial membrane potential, excessive ROS production and caspase cascade activation. Renal herb formula (RHF) reduces the apoptosis of resident renal cells and blunts inflammatory responses by targeting the p53-dependent intrinsic apoptotic pathway and nuclear factor kappa B (NF-κB)-mediated signalling (Tang et al., 2023). In the uninephrectomy (UNx) combined with high-purine diet model, compound probiotics inhibited the expression of B-cell lymphoma 2-associated X protein (Bax) and caspase-3 and terminal deoxynucleotidyl transferase dUTP nick end labeling (TUNEL)-positive cells to ameliorate renal mitochondrial injury and apoptosis (Liu P. et al., 2025).

Oxidative stress provides another major route linking urate exposure to apoptosis, with the ROS/hypoxia-inducible factor 1 alpha (HIF-1α)/enhancer of zeste homolog 2 (EZH2) axis playing a central regulatory role. UA facilitates the binding of HIF-1α to EZH2 and its promoter, enhancing EZH2 transcriptional activity; Jiangniaosuan formula inhibits this pathway to improve renal injury (Zhou et al., 2025). UA exposure also increases dynamin-related protein 1 (DRP1) expression and its phosphorylation at Ser616, promoting DRP1 mitochondrial translocation and interaction with mitochondrial fission 1 protein (FIS1), thereby enhancing mitochondrial fission, ROS overproduction, inflammation and apoptosis (Shen et al., 2025). In HUA, renal levels of inflammatory markers, apoptosis-related proteins, XO and 8-hydroxy-2′-deoxyguanosine are increased. However, these changes were attenuated by the presence of naringenin, which similarly reverses reduced glutathione (GSH) peroxidase activity (Calis et al., 2023). Besides these cell-autonomous mitochondrial and oxidative mechanisms, intercellular signalling also participates in apoptotic regulation. The exosomes secreted from urate-stimulated human renal tubular epithelial cells are capable of modulating inflammation, autophagy-related responses and apoptosis via the LincRNA-p21/allograft inflammatory factor 1 (AIF-1)/cytidine/uridine monophosphate kinase 2 (CMPK2)/NLRP3 signalling network (Hao et al., 2024), showing the complexity in intercellular regulatory activity in urate-associated injury.

Pharmacological interventions also have strong anti-apoptotic effects. Coptisine suppressed urate-induced apoptosis by downregulating cleaved caspase-3, apoptosis-inducing factor (AIF), cytochrome c, cleaved PARP and Bax and upregulating B-cell lymphoma 2 (Bcl-2) and phosphorylated Bad (Liu Y. et al., 2022). S-nitrosoglutathione, on the other hand, leads to mitochondrial dysfunction, oxidative stress and apoptosis in HK-2 cells via S-nitrosylation of peroxiredoxin 2 (PRDX2) (Han et al., 2025). Taken together, these data suggest that apoptosis is subject to orchestrated interactions between mitochondrial pathways and inflammatory signalling, but is additionally influenced by communication between cells, autophagy and ROS production. This integrated network supports a mechanistic basis and identifies potential targets for intervention in HUA-associated disorders.

### 3.2 Ferroptosis

HUA promotes ferroptosis through many converging mechanisms, but the most relevant to humans are increased oxidative stress by virtue of enhanced oxidative damage, dysregulated iron metabolism and impaired antioxidant defence. UA also promotes ROS production while inhibiting glutathione peroxidase 4 (GPX4), which aggravates doxorubicin (DOX)-induced ferroptotic cell death (Xu C. et al., 2024).

Moreover, urate induces intracellular Fe^2+^ accumulation in hepatocytes through a hypoxia-inducible factor 2 alpha (HIF-2α)-dependent upregulation of divalent metal transporter 1 (DMT1) associated with mitochondrial dysfunction and significant downregulation of the expression of GPX4, a hallmark feature closely related to the key ferroptosis execution phase (Wang T. et al., 2026). In foam cells, urate also inhibits mitochondrial function along with the inhibition of nuclear factor erythroid 2–related factor 2 (NRF2) and its downstream targets solute carrier family 7 member 11 (SLC7A11) and GPX4 (Yu et al., 2022). The use of Ferrostatin-1 (Fer-1), a pharmacological inhibitor of ferroptosis, not only ameliorates mitochondrial injury, but also restores NRF2/SLC7A11/GPX4 signalling and preserves foam cell viability impaired by urate (Yu et al., 2022), concomitantly with the activation of NRF2 by tert-butylhydroquinone (TBHQ), which reduces ER/mitochondrial stress caused by urate.

Iron homeostasis is also an essential regulator of urate-induced ferroptosis. HUA also upregulates nuclear receptor coactivator 4 (NCOA4), while downregulating SLC7A11, GPX4 and ferritin heavy chain 1 (FTH1) as markers of increased intracellular iron transportation on one hand and weakened antioxidant defences on the other (Zhang M. et al., 2025). Suppressor of cytokine signalling 1 (SOCS1) has also been implicated in ferroptosis. SOCS1 promotes the degradation of GPX4 by directly binding to GPX4 and leads to ubiquitin proteasome system driven GPX4 degradation (Zhang R. et al., 2025). On the other hand, natural product diosmetin (DIOS) has been shown to inhibit MSU crystal-induced ferroptosis in renal tubular epithelial cells through BCL2 interacting protein 3 (BNIP3)/NRF2/GPX4 activation that promotes NRF2 activation, GPX4 upregulation and augmented antioxidant defence (Hu C. et al., 2025).

Moreover, the results from *in vitro* and *in vivo* experiments demonstrate that Fer-1 increases the expression of SLC7A11, GPX4, ferritin light chain 1 (FTL1) and FTH1, but decreases intracellular malondialdehyde and iron levels, resulting in a marked improvement in HUA-related renal injury (Li Y. et al., 2024). These results emphasize targeting ferroptosis as a potential therapeutic strategy for preserving tissue integrity, inhibiting inflammation and restoring cellular homeostasis.

Mechanistically, urate causes ferroptosis by a pathogenic triad of increased ROS production, iron homeostasis dysregulation and antioxidant defence impairment, which together culminate in cell injury, inflammation and organ dysfunction. Ferroptosis-blocking strategies, including Fer-1 or NRF2 activators, provide a rational therapeutic approach to urate-associated pathology and a basis for the future exploration of ferroptosis in metabolic disease.

### 3.3 Pyroptosis

Pyroptosis is a type of pro-inflammatory PCD that occurs following inflammasome activation, caspase cleavage, generation of pores via the pore-forming protein GSDMD, and secretion of IL-1β and IL-18. Pyroptosis is at the centre of metabolic disturbance, inflammation and organ injury. Soluble urate-associated cellular stress can lead to pyroptotic signalling in HUA and, especially during crystal-mediated inflammation, phagocytosis of deposited MSU crystals may also elicit this.

In HUA, pyroptotic signalling should be interpreted according to the initiating urate species: soluble urate can enhance oxidative and organellar stress, whereas deposited MSU crystals undergo phagocytosis and strongly promote lysosomal destabilization and inflammasome activation. *In vivo* HUA-related injury is associated with NLRP3 inflammasome activation and pyroptotic cell death, as evidenced by caspase-1 and caspase-11 cleavage, GSDMD activation and IL-1β and IL-18 release, indicating the involvement of both canonical and non-canonical pathways in experimental models (Hu et al., 2022). Most importantly, mice rely on caspase-11 for the activation of non-canonical pyroptosis whereas humans depend mainly on caspase-4 and -5 in this pathway; therefore, findings related to caspase-11 should often be understood as preclinical studies rather than translated into human HUA directly. Upon phagocytosis of MSU crystals by macrophages, neutrophils or renal tubular epithelial cells, lysosomal destabilization occurs as a result of the release of cathepsins, and this in turn causes mitochondrial damage with associated production of ROS. Potassium efflux acts as an upstream signal to facilitate the formation of the NLRP3/apoptosis-associated speck-like protein containing a CARD (ASC)/pro-caspase-1 complex, leading to activation of caspase-1 by a downstream pathway. Inhibition of the NLRP3/caspase-1/GSDMD axis also supports a pivotal role for GSDMD-dependent pyroptosis in urate-linked tubular damage in HK-2 cells (Zhou et al., 2023). Activated caspase-1 cleaves pro-IL-1β and pro-IL-18 to generate mature IL-1β and IL-18 and simultaneously cleaves GSDMD to release its N-terminal pore-forming fragment. The process in turn increases membrane permeability, causing cell swelling and lysis as well as the release of pro-inflammatory mediators.

HUA-driven pyroptosis extends beyond gouty arthritis, with pronounced tissue specificity and pathway heterogeneity. MSU crystal deposition in the kidney can contribute to tubular epithelial injury, interstitial inflammation, extracellular matrix deposition and fibrosis through mechanisms involving inflammatory signalling, oxidative stress and pyroptosis. GSDMD has been proven to promote urate-induced tubular injury through the retinoic acid-inducible gene I (RIG-I)/caspase-1 pathway, providing a link with nucleic acid-sensing innate immune signalling and amplifying pyroptosis (Ma et al., 2023). Furthermore, activation of the caspase-11/GSDMD axis in renal tissue from murine HN models suggests that the non-canonical pyroptotic pathway contributes to experimental disease progression (Wu et al., 2024). HUA activates kidney inflammation-related PCD, which connects pyroptosis with aggravation of renal inflammation and tissue injury (Tian et al., 2026). Apart from NLRP3, the absent in melanoma 2 (AIM2) inflammasome may also be involved in inflammatory responses during acute gout and HUA in the absence of symptoms (Chu et al., 2024). The in vivo-based experiment also shows that inhibition of the NLRP3/caspase-1/GSDMD pathway reduces urate-induced hepatocyte pyroptosis and inflammation, which is suggestive of pyroptosis as a mechanism common to multi-organ inflammatory injury in the setting of hyperuricaemic states (Xu et al., 2025).

Pyroptosis is a significant inflammatory cell death mechanism in HUA-related tissue damage, with the NLRP3/caspase-1/GSDMD axis as the most consistently implicated core pathway and RIG-I, murine caspase-11, AIM2 and other context-specific signalling modules contributing to pyroptotic pathway heterogeneity. But the activation thresholds, temporal dynamics and causal interactions of pyroptosis with other PCD such as apoptosis, ferroptosis and autophagy-related dysfunctions are still ill-defined. Additional studies that take into account disease stage, tissue microenvironment and cellular heterogeneity will be required to clarify exactly how pyroptosis contributes to HUA-associated pathology.

### 3.4 Autophagy

Autophagy represents an important homeostatic mechanism that allows cells to respond to various challenges, including nutrient deprivation, oxidative stress, inflammatory stimuli and damaged organelles. Significantly, autophagy itself is not a cell death modality: even though intact autophagy is generally cytoprotective (and an impairment of autophagic flux may worsen the cellular injury), autophagy-dependent cell death must be reserved as a term when the components and/or functions involved in the execution of cell death are causally required for this process. It works via integrated mechanisms involving the formation of autophagosomes, lysosomal fusion and degradation of substrates to remove dysfunctional mitochondria, misfolded proteins and inflammation-related factors. Autophagy acts as a key regulatory junction connecting disordered urate metabolism, PCD and organ injury in HUA, whereby sustained increases in urate and MSU crystal deposition exert metabolic stress, mitochondrial uncoupling and inflammatory reactions.

Autophagy in the hyperuricaemic milieu controls biological responses in a non-linear manner that depends on stimulus strength, duration and cell type as well as autophagic flux status. Soluble urate or MSU crystals can increase intracellular ROS, decrease mitochondrial membrane potential and disrupt mitochondrial proteostasis and energy metabolism, thereby affecting pathways related to AMP-activated protein kinase (AMPK), NRF2 and mitophagy in a context-dependent manner. The inhibition of mitophagy by hydroxychloroquine (HCQ) further aggravates urate-induced endothelial injury, suggesting a vital role of mitochondrial quality control in cellular adaptation to urate stress (Wu et al., 2025). Mitophagy is an essential part of mitochondrial quality control; it is a process that eliminates damaged mitochondria and maintains vessel integrity by blocking sustained release of mitochondrial ROS and alleviating oxidative stress. Urate-induced oxidative stress causes direct damage to renal tubular epithelial cells and also stimulates extracellular matrix deposition and fibrotic remodelling through perturbation of antioxidant defences and autophagic activation. Therefore, targeting NRF2 with dioscin inhibits rather than promotes oxidative stress while stimulating protective autophagy to ameliorate fibrosis in urate-treated HK-2 cells, demonstrating the coordinated role of NRF2-regulated antioxidant responses and autophagy in cellular repair (Feng et al., 2025). The protective function of autophagy relies on both proper flux and degradation capacity. Chronic dysregulation of autophagy may result from sustained urate exposure transitioning from an adaptive, compensatory response to a pathogenic one. Prolonged dysregulation of autophagy may accompany irreversible loss of viability in PTECs and progression of tubulointerstitial fibrosis (Liu P. et al., 2025); however, this should be distinguished from autophagy-dependent cell death unless a causal requirement for the autophagic machinery is demonstrated. Therefore, the role of autophagy in HUA is not only dependent on its activation but is also determined by autophagic flux, effective removal of damaged mitochondria and cellular capacity to undergo reversible adaptive responses.

Urate-induced organ injury can be further influenced by dysregulated autophagy through its interplay with mitochondrial homeostasis, inflammasome activation and apoptotic pathways, thereby fostering pathological amplification at multiple levels. If damaged mitochondria are not quickly and efficiently removed, ROS continue to be released and propagate mitochondrial danger signals that lead to active NLRP3 inflammasome activation with increased secretion of IL-1β and IL-18, a switch from a transient metabolic stress response to persistent tissue damage. HUA promotes autophagy and NLRP3-dependent inflammatory processes that ultimately contribute to renal damage (Wu et al., 2021). Here it is essential that autophagic flux is intact for optimal mitochondrial removal and inflammatory amplification control. When the capacity to degrade misfolded proteins becomes impaired, oxidative stress, inflammasome activation and death signalling may enter into a self-propagating feedback loop. In models of diabetic nephropathy, empagliflozin prevents renal injury associated with concomitant HUA via AMPK-mediated autophagic induction and suppression of apoptosis (Zhang et al., 2026). The evidence suggests that AMPK-dependent autophagy provides renoprotection through restoration of energy homeostasis, preservation of mitochondrial function and restraint of caspase-induced apoptosis. Similar quality-control processes are employed in the setting of hepatic metabolic injury. Urate promotes clearance of damaged mitochondria through activation of PTEN-induced kinase 1 (PINK1)/Parkin-mediated mitophagy and subsequently mitigates dysregulation of lipid metabolism and prevents hepatic lipid accumulation (Chen et al., 2026a). However, the initiation of autophagy does not necessarily mean cytoprotection, because blockage in downstream degradative steps can lead to junk accumulation with augmented cellular stress. Urate-induced mitophagy occurs in cardiomyocytes with impaired autophagic flux, leading to p62 accumulation, defective mitochondrial turnover and persistent mitochondrial stress (Gao et al., 2021). Interventions directed at inflammatory signalling may help to break this maladaptive cycle. Electroacupuncture at BL23 mitigates urate-mediated renal inflammation and injury by inhibiting activation of the NLRP3 inflammasome and IL-6/Janus kinase (JAK)/STAT3 signalling (Hu Y. et al., 2025). Together, these data suggest that autophagy is not an autonomous degradative process but instead is a central regulatory node in networks of oxidative stress, inflammatory amplification, mitochondrial dysfunction and PCD. Mechanistically, these opposing behaviours depend on feedback with respect to the dynamics of autophagic flux, mitochondrial quality control and the relative extent to which inflammatory signalling is restrained.

The contribution of autophagy to HUA is largely context-dependent. Instead, intact autophagy at moderate levels preserves cellular homeostasis and limits the dissemination of inflammation, whereas impaired autophagic flux or sustained dysregulation can increase cellular stress, enhance susceptibility to regulated cell death and promote tissue remodelling. Thus, evaluation of autophagy should not solely rely on altered levels of markers like microtubule-associated protein 1 light chain 3 (LC3) or p62 but should combine these with measurements of autophagic flux in the context of disease progression and tissue-specific environments. Specific targeting of essential regulatory axes, such as AMPK, NRF2, PINK1/Parkin and NLRP3, may introduce novel therapeutic windows into the arena of urate-associated organ injury management. Relevant evidence is summarised in Table 1 to provide a systematic overview of the triggers, core signalling pathways and pathological roles of regulated cell death modalities and autophagy-related dysfunction in HUA.

**TABLE 1:** Regulatory mechanisms and roles of PCD in HUA.

PCD modality	Major triggers	Key regulators/pathways	Cellular features	Relationship to urate/MSU	Associated pathology	References
Pyroptosis	Urate	Autophagy–CTSB–NLRP3–GSDMD	Caspase-1/11 activation; IL-1β/IL-18 release	Autophagy-dependent NLRP3 pyroptosis	Renal fibrosis	(Hu et al., 2022)
Pyroptosis	Urate	ERK1/2–caspase-8/3–GSDME	Cell swelling; lactate dehydrogenase (LDH) release	GSDME cleavage via ERK1/2–caspase-3	Tubular injury	(Ding et al., 2022)
Pyroptosis	Urate	NLRP3–caspase-1–GSDMD	Membrane pores; IL-1β/IL-18 release	Canonical inflammasome activation	Renal inflammation	(Zhou et al., 2023)
Pyroptosis	Urate	RIG-I–caspase-1–GSDMD	GSDMD cleavage; IL-1β release	RIG-I-dependent inflammasome activation	Renal fibrosis	(Ma et al., 2023)
Apoptosis	Urate	lincRNA-p21/AIF-1-associated signaling	Annexin V positivity	Enhanced tubular apoptotic susceptibility	Tubular injury	(Hao et al., 2024)
Apoptosis	Urate	PI3K/AKT–Bcl-2/Bax–caspase-3	TUNEL positivity; mitochondrial dysfunction	Mitochondrial apoptotic activation	Tubular injury	(Liu Y. et al., 2022)
Pyroptosis	MSU	ROS–NLRP3–caspase-1–GSDMD	LDH release; membrane rupture	Inflammasome-dependent pyroptotic activation	Tubulointerstitial inflammation	(Liu et al., 2021)
Pyroptosis	MSU	Pannexin 1 (Panx-1)/P2X purinoceptor 7 (P2X7)–NLRP3–GSDMD	Membrane damage; LDH release	P2X7/Panx-1-dependent pyroptotic activation	Tubular epithelial injury	(Fu et al., 2024)
Apoptosis	Urate	SNO-PRDX2–ROS–mitochondrial pathway	ROS accumulation; TUNEL positivity	PRDX2-dependent mitochondrial apoptosis	Renal fibrosis	(Ha et al., 2025)
Apoptosis	Urate	HIF-1α–microRNA-295 (miR-295)–FIH-1	TUNEL positivity; caspase-3 activation	miR-295-dependent apoptotic regulation	Tubular injury	(Li et al., 2026)
Pyroptosis	Urate	NLRP3–ASC–caspase-1–GSDMD	LDH and IL-1β/IL-18 release	NLRP3-dependent pyroptotic injury	Tubular epithelial injury	(Zheng et al., 2023)
Apoptosis	HUA	SIRT1/6–NRF2; Bcl-2/caspases	TUNEL positivity; apoptotic protein changes	SIRT1/6–NRF2-associated apoptotic regulation	Tubular injury	(Yu et al., 2024)
Ferroptosis	Urate	SLC7A11–GPX4	Iron accumulation; lipid peroxidation	Suppression of the SLC7A11–GPX4 defence	Renal fibrosis	(Li Y. et al., 2024)
Pyroptosis	MSU	NADPH oxidase 1 (NOX1)–TLR4–NLRP3	GSDMD cleavage; IL-1β release	NOX1-dependent TLR4/NLRP3 activation	Renal inflammation	(Zhang Y. X. et al., 2025)
Apoptosis	HUA	ER stress–CHOP–Bax/Bcl-2	TUNEL positivity; ER stress	ER stress-driven apoptotic activation	Renal fibrosis	(Liu H. et al., 2025)
Ferroptosis	Urate	SOCS1–GPX4 ubiquitination	Mitochondrial damage; ferroptotic cell death	GPX4 degradation-dependent ferroptotic activation	Renal tubular injury	(Zhang R. et al., 2025)
Ferroptosis	Urate	NCOA4–NRF2–GPX4	Iron accumulation; lipid peroxidation	Ferritinophagy-dependent ferroptotic activation	Tubulointerstitial fibrosis	(Wang M. et al., 2026)
Necroptosis	HUA	Receptor-interacting serine/threonine-protein kinase 1 (RIPK1)–receptor-interacting serine/threonine-protein kinase 3 (RIPK3)–mixed lineage kinase domain-like protein (MLKL)	MLKL phosphorylation; loss of viability	RIPK1/RIPK3/MLKL-dependent necroptotic injury	Renal fibrosis	(Chen et al., 2026b)
Apoptosis	Urate	DRP1–FIS1 mitochondrial fission	Mitochondrial fragmentation; Annexin V positivity	DRP1–FIS1-dependent mitochondrial apoptosis	Tubular epithelial injury	(Shen et al., 2025)

## 4 Integrated signalling and crosstalk of PCD

Injury to tissues related to HUA results from orchestrated interactions among multi-layered signalling networks, such as oxidative stress, inflammatory response and metabolic dysregulation. In this model, different types of PCD are intertwined through intricate crosstalk, together orchestrating an inflammatory amplification loop. These signalling pathways are integrated and reciprocally regulated to drive the initiation and progression of organ injury, as summarised in Figure 3.

**FIGURE 3:**
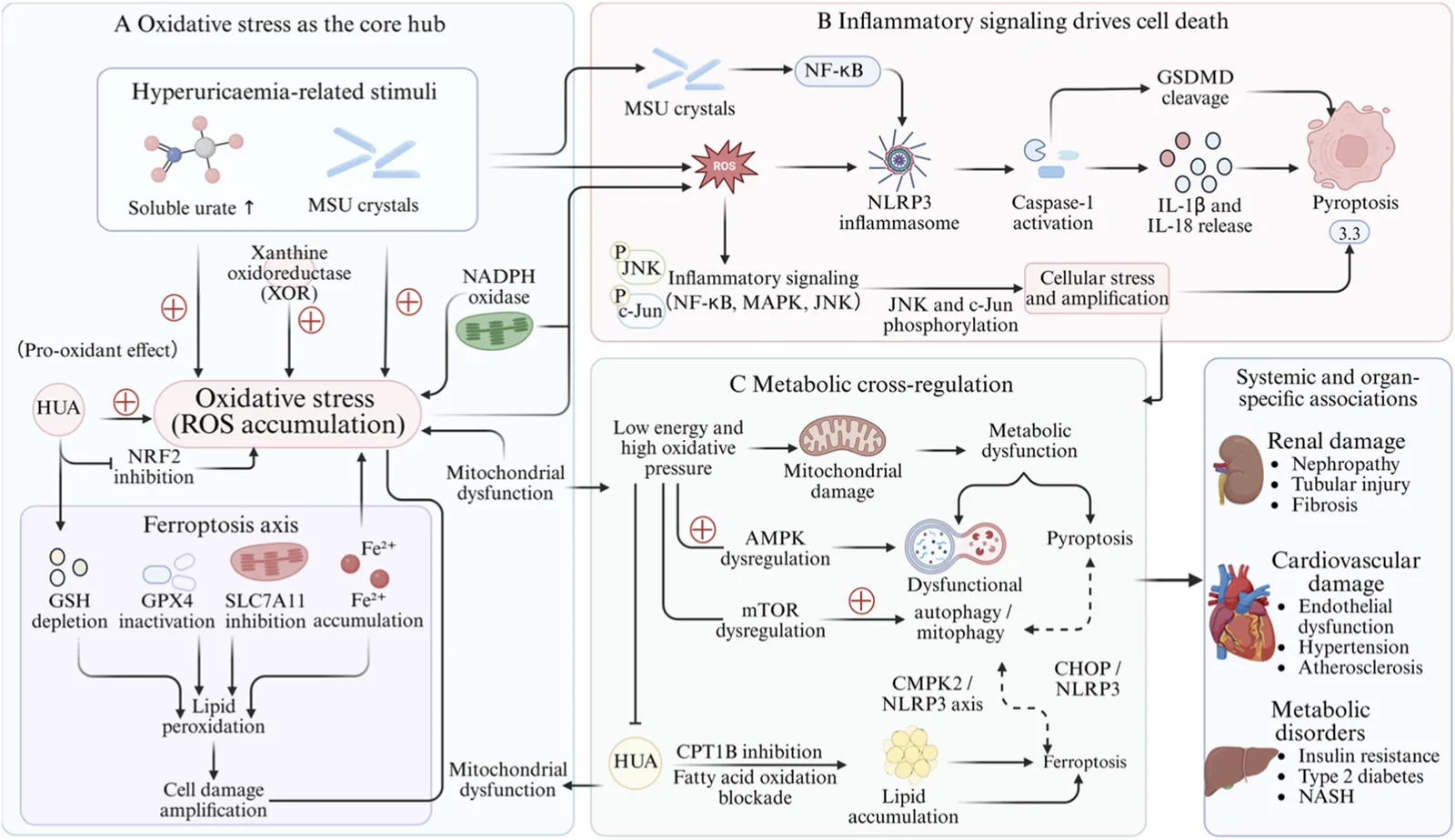
Integrated signalling and crosstalk of PCD in HUA: **(A)** Conceptually, oxidative stress is a central hub of elevated urate-associated ROS, compromised antioxidant defence systems and ferroptosis in HUA. **(B)** Activation of signalling related to NF-κB, MAPK and NLRP3 enhances the inflammatory response through pyroptosis mediated by caspase-1/GSDMD, which promotes further amplification of inflammation. **(C)** Defective autophagy/mitophagy and deranged lipid metabolism interact with altered AMPK/mTOR signalling, metabolic dysregulation and mitochondrial dysfunction. Crosstalk between these processes perpetuates oxidative, inflammatory and metabolic injury and mediates renal, cardiovascular and metabolic sequelae of HUA.

### 4.1 Oxidative stress as a central hub

Ferroptosis is an iron-dependent form of regulated cell death, distinctively characterized by lipid peroxidation and integral to disorders with disrupted iron metabolism, including mitochondrial dysfunction, augmented oxidative stress and activation of inflammatory networks. In osteoarthritis and HUA-associated tissue injury, cells are perpetually stimulated by inflammation, metabolic stress caused by hypoxia/ischemia and accumulated oxidative damage; this environment results in progressive conversion of homeostatic maintenance to a state of a self-amplifying loop.

In the osteoarthritic microenvironment, chondrocytes are persistently subjected to aberrant mechanical loading and inflammatory stimuli. This induces continuous lipid peroxidation via the Fenton reaction, generating large amounts of ROS from accumulation of non-transferrin-bound Fe^2+^. Glutathione depletion, compromised GPX4 activity and inhibited SLC7A11 function lead to more extensive accumulation of lipid peroxides, which promotes ferroptotic cell death. Cellular damage associated with HUA shows a similar oxidative injury paradigm. The effects of UA on macrophage-derived foam cells are associated with mitochondrial dysfunction leading to decreased cell viability and glutathione levels, elevated lipid ROS and iron deposition (Yu et al., 2022). High urate induces an increase in ROS generation and oxidative stress in NRK-52E cells (Qiao et al., 2023), and also exacerbates inflammatory and oxidative damage by inhibiting the NRF2 pathway (Chen et al., 2023). When the function of chondrocytes degenerates, type II collagen and aggrecan production is downregulated while matrix-degrading enzymes such as MMP-13, MMP-3, a disintegrin and metalloproteinase with thrombospondin motifs 4 (ADAMTS-4) and a disintegrin and metalloproteinase with thrombospondin motifs 5 (ADAMTS-5) are activated. Such an imbalance finally leads to disturbances of the collagen network, stimulates proteoglycan turnover and promotes progressive degeneration of the cartilage matrix.

HUA interacts with renal oxidative stress, tubular epithelial cell apoptosis and fibrotic responses and plays a significant contributory role in promoting the development of urate nephropathy (Feng et al., 2025). Therefore, persistent elevation of serum urate may lead to inflammation and damage of renal tubular epithelial cells. This is correlated with activation of NLRP3 inflammasome and apoptosis in HK-2 cells (Zheng et al., 2023). In addition, urate-induced oxidative stress promotes partial epithelial-to-mesenchymal transition in renal tubular cells and aggravates tubulointerstitial fibrosis in hyperuricaemic kidney disease (Cao et al., 2024).

Ferroptosis links cell death to matrix degradation in degenerative skeletal diseases, implying that a common mechanism underlies osteoarthritis and HUA-associated joint tissue damage. This relationship between impaired iron handling, lipid peroxidation, mitochondrial dysfunction and inflammatory amplification is well established and highlights the breakpoint at which adaptability converges into irreversible cell damage. Ferroptosis has been implicated in a variety of diseases; therefore, insight into targeting ferroptosis-related pathways may provide a therapeutic approach to alleviate cartilage matrix degradation and organ injury associated with HUA.

### 4.2 Inflammatory signalling drives cell death

Urate-associated tissue injury is not merely attributable to crystal deposition or metabolic imbalance but manifests as the ultimate result of the long-standing interplay among chronic inflammation, oxidative stress, mitochondrial dysfunction and PCD. Continuous exposure to urate increases the inflammatory and oxidative stress levels of vascular endothelial cells, renal tubular epithelial cells and immune cells, leading to a loss of cellular homeostasis and thus a change in NLRP3 inflammasome status as a central amplification point (Li X.-Q. et al., 2024).

Chronic exposure to urate leads to chronic activation of inflammatory pathways, including NF-κB, mitogen-activated protein kinase (MAPK) and JAK/STAT, thereby keeping the cells in a state of constant stress. MSU crystals trigger NF-κB signalling and induce ROS generation, leading to inflammasome activation and cytokine secretion in human umbilical vein endothelial cells (HUVECs) (Zhou et al., 2022). Importantly, MSU-induced inflammatory responses in fact rely on NLRP3 inflammasome activation (He M. et al., 2022). Moreover, MSU crystals induce JUN and phosphorylated JUN (p-JUN) in a JNK-dependent manner, which is required both for inflammatory activation and metabolic transcriptional reprogramming (Cobo et al., 2022). In this context, with persistent ROS accumulation, loss of mitochondrial membrane potential and increased lipid peroxidation, a vicious cycle between inflammatory amplification and oxidative damage is established, leading to progressive cellular dysfunction.

Sustained activation of the NLRP3 inflammasome induces caspase-1 activation and the maturation and secretion of IL-1β and IL-18, ultimately driving pyroptosis, which is a canonical inflammatory form of PCD (Ahn et al., 2021). It has been shown that NLRP3/caspase-1/GSDMD axis activation is involved in urate-induced pyroptosis in HK-2 cells (Zhou et al., 2023). In addition to pyroptosis, dysregulated autophagy and NLRP3-dependent inflammation also mediate renal injury exacerbation in HUA (Wu et al., 2021). Concurrently, urate-associated gut microbiota dysbiosis might aggravate renal injury by enhancing the generation of gut-derived uremic toxins, which subsequently upregulate NLRP3 inflammasome activation (Zhou et al., 2024). These various processes create a toxic cycle that results in an increasingly interconnected pathological network connecting inflammation, metabolic dysfunction and PCD.

Mechanistic details identified through research have shown that inflammation-driven PCD is a core pathophysiological mechanism underlying the progressive tissue damage associated with HUA. Overall, targeting a few key nodes including NF-κB signalling, NLRP3 inflammasome activation, ROS generation and downstream cell death pathways may open up new strategies to ameliorate renal and vascular injury. Studies in the future should address how inflammatory signalling and its interplay with cell death are defined at different stages of disease to yield therapeutic strategies that modulate injury repair.

### 4.3 Crosstalk between metabolism and cell death pathways

HUA-associated tissue injury is not a consequence of a singular metabolic derangement or cell death pathway, but represents the complex interaction between dysregulated energy metabolism, oxidative stress, chronic inflammation and multiple pathways of PCD. Mitochondrial dysfunction, lipid metabolic imbalance and impaired antioxidant capacity occurring in the hyperuricaemic milieu shift the equilibrium between adaptive compensation and irreversible injury towards a pathological outcome. Importantly, ferroptosis is intimately linked to dysregulated autophagy and inflammatory amplification along this pathway.

Chondrocytes under persistent energy deficiency and elevated oxidative stress manifest decreased efficiency of mitochondrial oxidative phosphorylation and ATP production while exhibiting leakiness from the electron transport chain that results in chronic accumulation of ROS. This enhances Fe^2+^-dependent Fenton reactions and increases lipid peroxidation. The CMPK2/NLRP3 axis is also important in the amplification of inflammation, and CMPK2 activates NLRP3 to promote the inflammatory response and pyroptosis (Hao et al., 2024). Additionally, urate can directly target β-cells through the C/EBP homologous protein (CHOP)/NLRP3 pathway (Li et al., 2025). Mitochondrial homeostasis and antioxidant abilities gradually degrade as disruption within cellular energy-sensing systems continues. They display accumulated oxidative damage due to the insufficient clearance of impaired mitochondria, indicating a shift from metabolic adaptation of chondrocytes towards greater susceptibility to ferroptosis. Importantly, the tissue injury associated with HUA retains some of the characteristics of osteoarthritis, which include metabolic derangement, oxidative stress and apoptosis, highlighting that mechanisms with implications for metabolic and cell death crosstalk in diseases like osteoarthritis may also inform a greater understanding of hyperuricaemic conditions.

In this respect, mitophagy together with lipid metabolic homeostasis exerts critical protective roles. Mitophagy specifically eliminates damaged mitochondria from cells in order to conserve energy and metabolic homeostasis to keep cellular functions intact (Wu et al., 2025). UA has been demonstrated to induce mitophagy in cardiomyocytes through the ROS/CaMKIIδ/Parkin pathway (Gao et al., 2021); however, this cytoprotective effect may diminish after prolonged metabolic dysregulation. Urate may also induce apoptosis in foam cells via autophagy suppression (Liu W. et al., 2023). In keeping with these findings, knockdown of PINK1 consistently hinders mitophagy, which further promotes CD36 upregulation and results in increased lipid accumulation (Chen et al., 2026a). Coinciding with this, targeted metabolomic analyses in gouty arthritis have demonstrated extreme lipid and lipid-like accumulation, which leads to impaired autophagic flux that can also affect inflammatory and immune responses (Fu et al., 2023). In addition, urate can inhibit the activity of carnitine palmitoyltransferase 1B (CPT1B) and restrict fatty acid transport into mitochondria for β-oxidation (Yang et al., 2023). This leads to impaired fatty acid oxidation, lipid excess, as well as prolonged oxidative stress, leading to a vicious intracellular cycle of lipid peroxidation, matrix catabolism and ferroptotic cell death over time.

The failed crosstalk between metabolic dysfunction and ferroptosis emphasizes that tissue injury induced by HUA is not a single-mode-related form of cell death but an outcome of the convergence of energy imbalance, lipid dysregulation, compromised antioxidant defence and perpetual inflammatory activation. Focusing on crucial regulatory pathways such as AMPK, NRF2, GPX4 and autophagy, including mitophagy and lipid catabolism, may provide new possibilities in organ injury protection and metabolic restoration during HUA. However, the precise regulatory mechanisms controlling these interactions are still to be uncovered.

## 5 PCD in HUA-associated diseases

### 5.1 Gout

Gout is the main clinical manifestation of HUA and results from the deposition of MSU crystals in joints and adjacent tissues. As plasma urate levels reach the solubility threshold, MSU crystals activate innate immune receptors such as NLRP3 and initiate acute inflammatory responses (Bai et al., 2022). PCD is a central regulator of the initiation and maintenance of gouty inflammation. Pyroptosis is the major regulated cell death pathway, while apoptosis and neutrophil extracellular trap (NET) formation contribute to inflammatory cell injury and autophagy acts as a context-dependent regulator of inflammasome activity and cellular homeostasis. The role and regulatory networks of PCD in diseases related to HUA are systematically summarised in Table 2. These disease-specific PCD networks and their convergence on shared inflammatory, oxidative and metabolic injury are illustrated in Figure 4.

**TABLE 2:** Regulatory mechanisms and roles of PCD and related processes in HUA-associated diseases.

HUA-associated disease	PCD modalities and related processes	Principal cell types/tissues	Key regulators/signalling pathways	Pathophysiological roles	Clinical relevance/potential therapeutic targets	References
HN/CKD progression	Pyroptosis-related mechanisms; caspase-11–GSDMD-dependent NET formation	Neutrophils, macrophages and renal interstitium (cell + animal models)	Caspase-11–GSDMD axis; NETs; IL-1β, TNF-α, transforming growth factor beta 1 (TGF-β1), HMGB1; α-SMA, collagen I; TGF-β1/p-Smad3	Promotes inflammatory cell infiltration, NET formation and profibrotic signalling, driving macrophage-to-myofibroblast transition and interstitial fibrosis	Caspase-11–GSDMD-dependent NET formation as a potential therapeutic target	(Wu et al., 2024)
Urate-induced tubular injury	GSDMD-mediated pyroptosis	Renal tubular epithelial cells; kidney (cell + animal models)	RIG-I–caspase-1–GSDMD	Promotes pyroptosis, inflammation and tubular injury	RIG-I–caspase-1–GSDMD axis as a potential therapeutic target	(Ma et al., 2023)
Urate-induced renal injury	Pyroptosis and apoptosis	Renal tubular epithelial cells; kidney (cell + animal models)	NOX1; TLR4–MyD88–NF-κB; NLRP3–caspase-1–GSDMD; Bcl-2 pathway; ROS	Enhances oxidative stress, inflammation and cell death, leading to tubular injury and fibrosis	NOX1–TLR4/NLRP3 signalling as a candidate therapeutic target	(Zhang Y. X. et al., 2025)
Gouty arthritis (MSU-induced acute inflammation)	Pyroptosis	Macrophages and synovial tissue (cell + animal models)	NRF2–mitochondrial reactive oxygen species (mtROS)–NLRP3 inflammasome; NEK7; GSDMD	MSU activates inflammasomes, inducing macrophage pyroptosis and IL-1β release, driving joint inflammation	NRF2/mtROS/NLRP3 signalling as a potential intervention target	(Lin et al., 2020)
Gouty arthritis	Pyroptosis	Macrophages and synovial tissue (cell + animal models)	bromodomain-containing protein 4 (BRD4)–MDM2 proto-oncogene (MDM2)–PPARγ axis; NLRP3 inflammasome	MSU-induced PPARγ degradation enhances inflammasome activation and pyroptosis	BRD4–MDM2–PPARγ axis as a candidate intervention target	(Xu and Qiu, 2024)
MSU-induced acute gout	Pyroptosis	Macrophages and joint tissue (cell + animal models)	ROS–thioredoxin-interacting protein (TXNIP)–NLRP3 pathway	ROS-driven inflammasome activation promotes pyroptosis and inflammation	ROS–TXNIP–NLRP3 inhibition as a potential anti-inflammatory strategy	(Luo et al., 2022)
HUA-associated liver injury	Apoptosis (mitochondrial)	Hepatocytes and liver tissue (cell + animal models)	Phosphatidylserine decarboxylase (PISD)-mediated phospholipid metabolism; STAT3–Bcl-2; Bax; caspase-3	Disrupts mitochondrial lipid homeostasis, increases ROS and apoptosis	PISD-mediated mitochondrial lipid metabolism as a potential hepatoprotective target	(Liu N. et al., 2023)
Diabetic nephropathy with hyperuricemia	Apoptosis; autophagy	Renal tubular epithelial cells; kidney (cell + animal models + patient renal tissue samples)	AMPK pathway; LC3; caspase-3; URAT1	Reduced autophagy and increased apoptosis exacerbate fibrosis	AMPK activation as a potential therapeutic strategy	(Zhang et al., 2026)
HUA-associated atherosclerosis	Ferroptosis; autophagy dysfunction	Foam cells and atherosclerotic plaques (cell + animal models)	NRF2/SLC7A11/GPX4 axis; lipid ROS; Fe^2+^	Promotes lipid peroxidation, iron overload and plaque progression	Ferroptosis inhibition and NRF2 activation as potential intervention strategies	(Yu et al., 2022)
HUA-associated liver injury	Ferroptosis	Hepatocytes and liver tissue (cell + animal models)	HIF-2α–DMT1 axis; NOX4; GPX4	Induces iron overload, oxidative stress and mitochondrial dysfunction	HIF-2α/DMT1 signalling and ferroptosis as candidate therapeutic targets	(Wang T. et al., 2026)
Urate-induced tubular injury	Pyroptosis and mitochondrial apoptosis	Renal tubular epithelial cells and kidney (cell + animal models)	NLRP3–caspase-1–GSDMD; Bcl-2/Bax; URAT1, GLUT9	Concurrent pyroptosis and apoptosis exacerbate tubular injury	Combined modulation of PCD and urate transport as a potential therapeutic strategy	(Wang et al., 2021)
HUA with acute gout/renal dysfunction	Pyroptosis	HK-2 cells, macrophages, joint and kidney tissue (cell + animal models)	ROS–NEK7–NLRP3 axis; ABCG2; XOD	Promotes inflammasome activation, IL-1β release and tissue injury	NEK7–NLRP3 inhibition as a potential therapeutic strategy	(Zhao Z. et al., 2025)
HUA-associated renal injury	Pyroptosis	Renal tubular epithelial cells and kidney (cell + animal models)	AMPK–TLR4–NF-κB–NLRP3 pathway; urate transporters	Reduced AMPK activity enhances inflammasome signalling and pyroptosis	AMPK activation as a potential therapeutic strategy	(Tian et al., 2026)
CKD and fibrosis (HUA-related)	Autophagy	Renal tubular cells and kidney (cell + animal models)	Autophagy/ATG5; TGF-β–NLRP3–EMT axis	Autophagy restrains inflammasome activation and fibrosis	Autophagy induction and NLRP3 inhibition as a potential antifibrotic strategy	(Wang et al., 2020)
Acute gout (MSU-induced)	Pyroptosis	Synovial macrophages and joint tissue (cell + animal models)	P2Y14 receptor (P2Y14R)–cyclic adenosine monophosphate (cAMP)–NLRP3 pathway	Reduced cAMP enhances inflammasome activation and inflammation	P2Y14R/cAMP signalling as a candidate target for acute gout	(Li et al., 2020)
HUA-aggravated NASH	Pyroptosis	Hepatocytes and liver tissue (cell + animal models)	GLUT9-mediated urate uptake; NLRP3–caspase-1–GSDMD	Promotes hepatic inflammation, steatosis and fibrosis	Urate transport and NLRP3 signalling as potential intervention targets	(Xu et al., 2025)
Endothelial injury (HUA-related)	Apoptosis	HUVECs (cell model)	NRF2–HO-1/NQO1; Bcl-2/Bax; ROS	Induces oxidative stress and endothelial apoptosis	NRF2 activation as a potential endothelial-protective strategy	(Chen et al., 2023)
Endothelial injury	Apoptosis	Vascular endothelial cells; aortic endothelium (cell + animal models)	C1q/TNF-related protein 3 (CTRP3)–TLR4 axis; oxidative stress pathways	Promotes inflammation, oxidative stress and endothelial apoptosis	CTRP3–TLR4 signalling as a potential therapeutic target	(Zhang et al., 2022)
HUA-associated renal injury	GSDME-mediated pyroptosis	NRK-52E tubular epithelial cells (cell model)	ERK1/2–caspase-8/3–NLRP3–GSDME	Induces membrane damage and inflammatory cell death	ERK1/2–caspase-8/3–GSDME axis as a candidate therapeutic target	(Ding et al., 2022)
HUA-induced erectile dysfunction	Apoptosis	Penile and renal tissues (animal model)	NLRP3 inflammasome; Janus kinase 2 (JAK2)/STAT3 pathway	Promotes apoptosis, oxidative stress and inflammation, reducing NO and testosterone	NLRP3 and JAK2/STAT3 signalling as potential intervention targets	(Ge et al., 2024)

**FIGURE 4:**
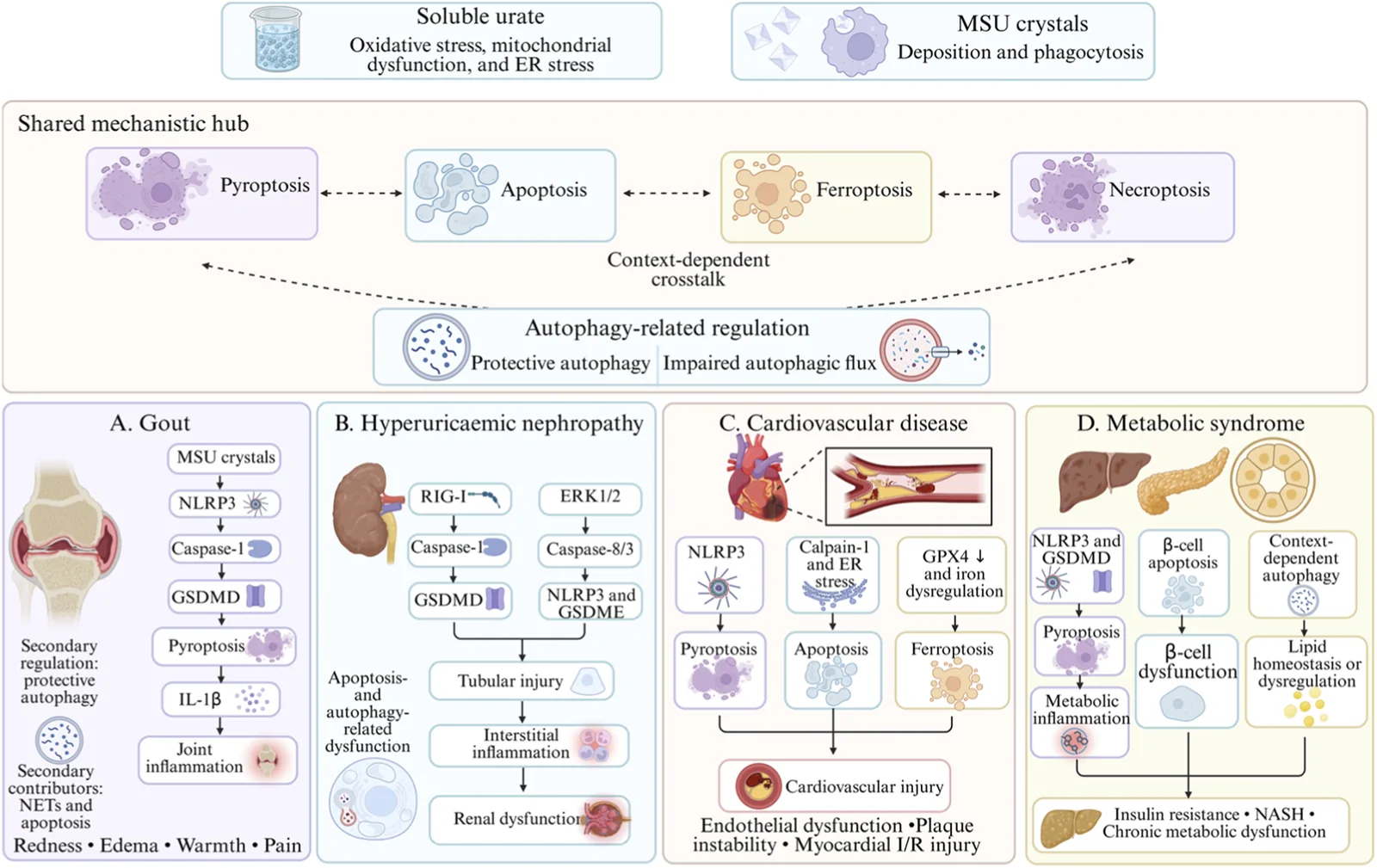
PCD crosstalk in HUA-associated diseases: Soluble urate and MSU crystals activate overlapping oxidative, inflammatory and metabolic injury pathways in different HUA-associated diseases. **(A)** In gout, MSU crystal-induced inflammasome activation and pyroptosis predominate, with additional contributions from apoptosis, NETosis and autophagy-related processes. **(B)** In HN, various pyroptotic pathways, apoptosis and autophagy-associated dysfunction contribute to tubular injury and renal inflammation. **(C)** In cardiovascular disease, pyroptosis, apoptosis, ferroptosis and autophagy dysfunction contribute to endothelial and myocardial injury. **(D)** In metabolic syndrome, pyroptosis, apoptosis and context-dependent autophagy are linked to IR, lipid dysregulation and chronic metabolic inflammation.

The NLRP3 inflammasome is the core molecular platform mediating MSU-induced pyroptosis in gouty inflammation. MSU crystals initiate pyroptosis of neutrophils and macrophages by triggering NLRP3-dependent caspase-1 activation, GSDMD cleavage and generation of the active pore-forming N-terminal fragment of GSDMD (N-GSDMD) (Chen et al., 2024). This is paralleled by strong discharge of pro-inflammatory mediators, among which is IL-1β, that aggravate local irritation and support the trademark clinical signs of joint redness, edema, warmth and pain. Mechanistically, DEAD-box helicase 3 X-linked (DDX3X) has been demonstrated to aggravate gouty inflammation via the activation of the NLRP3 inflammasome and pyroptosis in macrophages (Zhong et al., 2025). At the same time, IL-1A promotes MSU-induced apoptosis and inflammatory responses through Toll-like receptor 4 (TLR4)/myeloid differentiation primary response 88 (MyD88)/NF-κB signalling (Pan et al., 2025).

Besides inflammasome activation, oxidative stress and autophagy serve as two major regulators of PCD in gouty disease. Aldehyde dehydrogenase 2 (ALDH2) promotes NRF2 nuclear translocation and suppresses oxidative stress and inflammation by reducing the generation of ROS and inhibiting the assembly of the NLRP3 inflammasome and IL-1β release in acute gouty arthritis to mitigate damage (Tong et al., 2025). Autophagy counteracts inflammatory responses by promoting the degradation of various inflammasome components and clearing damaged organelles to control hyper-inflammation and maintain cellular homeostasis and the intracellular environment. PP121-induced autophagy is known to promote NLRP3 inflammasome degradation (Yuan et al., 2023), whereas Qingre Huazhuo Jiangsuan Decoction-mediated inhibition of the phosphoinositide 3-kinase (PI3K)/protein kinase B (AKT)/mechanistic target of rapamycin (mTOR) pathway stimulates autophagy and alleviates MSU-induced inflammatory injury (Liu P. et al., 2023). A study of Gnetum montanum extract (GME) also supports this therapeutic approach, confirming that it protects macrophages from MSU-induced pyroptotic injury (Pham et al., 2026).

Gouty inflammation is also regulated by other types of cell death, such as neutrophil extracellular trap formation (NETosis) and apoptosis. A possible therapeutic approach for acute gouty arthritis may be a drug aimed at modulation of NETosis versus apoptosis through targeting of GPR105 (Liu C. et al., 2022). In summary, PCD in gout is highly dependent on MSU-induced inflammatory signalling and, hence, many different mechanisms of cell death function in unison to potentiate local inflammation and tissue damage. Of them, pyroptosis mediated by the NLRP3 inflammasome has been the most characterised mechanism. It is expected that more detailed methods of characterization and delineation of signalling networks related to PCD will enhance the understanding of urate-induced inflammatory injury, possibly revealing further opportunities for therapeutic targets.

### 5.2 HN

HN is an important complication of HUA, which manifests as tubular injury, interstitial inflammation and progressive deterioration of renal function. Apart from MSU crystal deposition, oxidative stress and inflammatory responses are increasingly recognised as important mechanisms underlying disease progression. Moreover, pyroptosis has recently been the focus of much interest due to its strong pro-inflammatory properties. Simultaneously, apoptosis and other regulated cell death pathways, together with dysregulated autophagy, contribute to urate-induced kidney injury and are closely related to tubular epithelial cell damage, persistent inflammatory activation and kidney dysfunction.

In HN, tubular pyroptosis is intimately connected to activation of inflammasome-associated signalling pathways. On the other hand, HUA has been reported to activate the RIG-I/caspase-1/GSDMD axis in renal tubules, inducing inflammatory cell death, and genetic ablation of GSDMD markedly impairs renal dysfunction following HN (Ma et al., 2023). Neferine inhibits macrophage M1 polarisation and pyroptosis via the NLRC5/NLRP3 pathway, thereby reducing inflammation-associated renal injury (Yin et al., 2025). Likewise, fucoidan (FPS) ameliorates urate-induced NLRP3-dependent pyroptosis in HK-2 cells via inhibition of NF-κB signalling (Tan et al., 2024). Moreover, gasdermin E (GSDME)-mediated pyroptosis participates in urate-induced tubular injury and the total extract of Abelmoschus manihot L. flower (TEA) could ameliorate this process by inhibition of the extracellular signal-regulated kinase 1/2 (ERK1/2)/caspase-8/caspase-3/NLRP3/GSDME axis (Ding et al., 2022). Taken together, these results suggest that several pyroptotic pathways play a role in HN.

Other key regulators of HN are apoptosis and autophagy. Coptisine mitigates urate-induced renal inflammation, oxidative stress and mitochondrial apoptosis through the modulation of the PI3K/AKT pathway (Liu Y. et al., 2022). Likewise, ginsenoside Rg5 plays an extensive protective role since it alleviates inflammation, oxidative stress and both pyroptosis and apoptosis via simultaneous inhibition of the activation of these different PCD modalities (Zhang Y. X. et al., 2025). Gout formula ameliorates cellular pyroptosis in HK-2 cells by modulating the AMPK/TLR4/NLRP3 axis, an effect that is further enhanced by 5-aminoimidazole-4-carboxamide 1-β-D-ribofuranoside, an AMPK agonist (Tian et al., 2026). Moreover, the lincRNA-p21/AIF-1/CMPK2/NLRP3 signalling module can be exported as an exosome to regulate urate-induced inflammation, autophagy-related responses and apoptosis in human renal tubular epithelial cells (Hao et al., 2024), further reinforcing that regulated cell death pathways and autophagy-related processes cooperate in HUA-mediated renal injury.

Therefore, taken together, these findings indicate that HN involves coordinated regulation of multiple cell death pathways, including pyroptosis and apoptosis, together with dysregulated autophagy. The combined actions of these processes are responsible for tubular injury, interstitial inflammation and the ultimate progression of renal dysfunction; among these processes, NLRP3 inflammasome mediated pyroptosis remains a central focus in current research. An exact insight into the timing and relationships between different PCD modalities in the context of renal injury will further optimize the understanding of the molecular pathogenesis of HUA-associated kidney damage, possibly leading to new strategies for targeting HN.

### 5.3 Cardiovascular disease

HUA is strongly associated with a range of cardiovascular diseases, including hypertension, atherosclerosis, coronary heart disease and heart failure. In addition to acting as a metabolic marker, urate plays an active role in promoting cardiovascular damage through oxidative stress, inflammation and ECD. More recently, evidence has emerged implicating PCD as a key contributor to cardiovascular pathology associated with urate. Injury to endothelial cells, vascular smooth muscle cells and cardiomyocytes involves multiple regulated cell death modalities, including pyroptosis, apoptosis and ferroptosis, together with dysregulated autophagy, and is closely associated with ongoing inflammatory activation, vascular remodelling and cardiac dysfunction.

Urate-induced endothelial and myocardial injury in the cardiovascular system is largely facilitated via ROS-dependent activation of the NLRP3 inflammasome. Increased soluble urate causes ROS production in cells and activates the NLRP3 inflammasome to promote pyroptosis of endothelial cells (He et al., 2023). In accelerated myocardial ischaemia reperfusion injury, the NLRP3-mediated inflammatory cascade and pyroptosis are also aggravated: urate-driven ROS generation promotes these processes (Shen et al., 2021). Chronic inflammatory signalling aggravates ECD, leading to localized inflammation and atherosclerotic disease progression. At the same time, HUA promotes phenotypic switching of vascular smooth muscle cells, an event that autophagy has also been suggested to regulate (Lu et al., 2024).

Apart from pyroptosis, apoptosis and ferroptosis also contribute to urate-induced cardiovascular injury, while dysregulated autophagy modulates cellular homeostasis and susceptibility to injury. Via the action of calpain-1 and ER stress, UA promotes cardiomyocyte apoptosis (Yan et al., 2018), and, through inhibiting autophagy in foam cells, facilitates foam cell apoptosis and enhances the instability of atherosclerotic plaques (Liu W. et al., 2023). Basal autophagy is a dominant contributor to cell homeostasis, but sustained HUA compromises autophagic functions and worsens cellular damage. Urate is a key player in atherosclerosis through promoting NRF2-related autophagy dysfunction and ferroptosis (Yu et al., 2022). Moreover, urate increases oxidative stress and inhibits the expression of GPX4, thereby enhancing DOX-induced ferroptosis (Xu C. et al., 2024). The ferroptosis inhibitors deferoxamine and Fer-1 reduce pathological damage in myocardial ischaemia reperfusion injury, indicating the roles of the NCOA4/FTH1 (Zhang M. et al., 2025), and xCT/GSH/GPX4 axes in HUA-exacerbated cardiac injury.

The pathological activation and crosstalk of cell death pathways regulated at multiple levels as well as autophagy-related processes occur during the evolution of cardiovascular injury in human patients and experimental models of HUA. Pyroptosis elicits propagating inflammatory cascades, while apoptosis and dysregulated autophagy impart homeostatic loss by different but interconnected processes. Ferroptosis aggravates myocardial and vascular injury through oxidative stress and impairment of iron metabolism. The molecular mechanism by which HUA precipitates CVD links NLRP3 inflammasome mediated pyroptosis as a central node. This suggests that deeper interrogation of key PCD signalling pathways may uncover novel targets for early intervention and precision therapies in urate-deranged cardiovascular disease.

### 5.4 MetS

MetS is a conglomerate of various metabolic aberrations such as obesity, insulin insensitivity, dysregulation of glucose and lipid metabolism, hypertension and HUA. HUA is not only a very frequent comorbidity, but also contributes to key pathogenic processes such as IR, lipid dysregulation and chronic low-grade inflammation. There is an important regulatory role of PCD in the development of MetS. Adipose tissue inflammation, pancreatic β-cell injury and disturbed metabolic homeostasis involve regulated cell death modalities such as pyroptosis and apoptosis, together with context-dependent autophagy, all of which are closely linked to oxidative stress, inflammatory amplification and metabolic disturbances.

The NLRP3 inflammasome integrates urate signalling with inflammatory stimuli and metabolic homeostasis in the environment of metabolic dysregulation. Urate stress in HUA could enhance NLRP3/caspase-1/GSDMD-dependent pyroptotic signalling that worsens non-alcoholic steatohepatitis (NASH) (Xu et al., 2025). Urate also stimulates the TLR4/NLRP3 pathway and NF-κB signalling connected with both intestinal epithelial damage and mitochondrial dysfunction, thus downregulating the tight junction proteins (Ma et al., 2020). Markedly, persistent inflammatory signalling provides a feed-forward mechanism that aggravates metabolic imbalance, promotes intestinal barrier dysfunction and sustains chronic low-grade inflammation, giving rise to pyroptosis-related properties essential for urate-driven metabolic injury.

Metabolic disturbances related to HUA are also affected by apoptosis. High urate impairs pancreatic β-cell function and contributes to oxidative stress and IR. In the context of type 2 diabetes, urate suppresses Min6 cell proliferation, promotes apoptosis and stimulates caspase-3 activity (Jiang et al., 2022). Palmitate-stimulated upregulation of URAT1 in cardiomyocytes activates the MAPK pathway and induces apoptosis, ROS production and inflammatory responses (Tanaka et al., 2023). Persistently high concentrations of UA worsen lipid accumulation and metabolic imbalance, thus negatively affecting insulin response and cellular energy metabolism.

Autophagy plays a context-dependent role in metabolic homeostasis. Basal autophagic activity can facilitate the clearance of damaged mitochondria and misfolded proteins, thereby maintaining lipid metabolism and energy homeostasis, alleviating inflammatory damage and improving IR. It has been demonstrated that UA increases hepatic autophagy and that activation of this pathway appears to protect against hepatic IR (He F. et al., 2022). However, in the context of chronic HUA, loss of autophagic function may aggravate lipid dysregulation and peripheral injury, suggesting that its overall effect is strongly dependent upon disease state, cell type and metabolic environment.

Dysregulated PCD and MetS collectively embody a multiple-hit theory of accumulated risk arising from chronic inflammatory activation, so-called cytostasis, loss of cellular homeostasis and impaired energy metabolism. Pyroptosis enforces urate-driven metabolic inflammation, apoptosis drives pancreatic β-cell and metabolic tissue death, and impaired autophagy leads to dysregulated lipid handling and IR. These regulated cell death pathways can interact with autophagy-related processes, and their exact relationships in MetS require further clarification. Identifying these mechanisms will narrow the understanding of urate-linked metabolic injury and identify novel therapeutic targets.

## 6 Therapeutic strategies and intervention targets

### 6.1 Urate-lowering therapy

Evidence suggests that a long-term course of urate-lowering therapy, besides controlling serum urate and crystal deposition, has specific effects on oxidative stress, inflammatory amplification, mitochondrial dysfunction and metabolic disorder. Chronic HUA exerts prolonged oxidative and inflammatory stress on a range of different cell types, causing a transition from homeostatic to pathological injury mediated by altered PCD. There is emerging evidence that urate-related signalling may be targeted therapeutically to beneficial effect. As a putative GPR35 agonist, isobavachin decreases urate-mediated bone loss and may represent a potential therapeutic agent for HUA-related metabolic bone injury (Cen et al., 2025).

Inhibiting XOR is one of the important strategies for decreasing urate generation. Furthermore, in experimental HUA, notopterol has been reported to alleviate HUA-associated cardiac dysfunction (Wang et al., 2023), and other metabolites from Indian Muscodor species possess XO-inhibitory and antioxidant capacities (Kapoor and Saxena, 2016). These agents limit urate production and inhibit XO-derived ROS, resulting in alleviation of not only mitochondrial oxidative injury, but also lipid peroxidation and membrane damage. Decreasing oxidative stress and stabilizing mitochondrial function will prevent membrane potential loss, reduce cytochrome c release and subsequent caspase activation, and thus apoptosis. Concurrently, decreased oxidative signalling inhibits NLRP3 inflammasome activation, IL-1β release and gasdermin pore formation to alleviate pyroptosis and propagation of inflammation. This may explain why natural compounds can have complementary effects. For example, linarin attenuates HN by modulating NRF2/KEAP1 and TLR4/NF-κB signalling pathways (Qian et al., 2025), indicating that bioactive molecules may alleviate urate-associated oxidative and inflammatory injury through multi-pathway regulation.

Improving urate excretion provides an alternative benefit to the treatment of HUA. Dotinurad selectively inhibits URAT1 and reduces renal tubular reabsorption, decreasing serum urate (Yanai et al., 2025). With declining urate burden, inflammatory stress related to chronic HUA is relieved and dysregulation of metabolic sensing pathways, such as AMPK, mTOR, sirtuin 1 (SIRT1) and HIF-1α, could progressively normalise, restoring mitophagy, energy metabolism and ER homeostasis. At higher doses, quercetin promotes urate excretion and decreases crystal deposition, indicating a greater capacity of renal clearance (Liu H. et al., 2025). On the other hand, uricolytic bacteria in probiotics also reduce serum urate in animal models and exhibit beneficial effects on haemodynamics and renal function, suggesting that gut microbiota dysbiosis might be a valuable adjunctive approach for lowering serum urate. Other lifestyle interventions ameliorating serum urate and oxidative balance include decreased alcohol intake, increased hydration and combined pharmacotherapy.

Urate-lowering therapy provides an obvious point of entry for modulating PCD in diseases associated with HUA. Yet diminished circulating urate alone may be inadequate to restore established networks of inflammation, mitochondrial dysfunction or chronic tissue remodelling. In chronic pathological settings, some cell death pathways may remain activated, and different urate-lowering agents may exert specific effects on apoptosis, pyroptosis, necroptosis and ferroptosis, as well as on autophagic homeostasis. Consequently, future therapy should not only aim at the control of urate levels but also include strategies for restoring tissue homeostasis, optimising the metabolic environment surrounding cells and inhibiting disease progression over the long term. The therapeutic logic linking HUA-driven pathogenic mechanisms, PCD-targeted interventions and restoration of organ homeostasis is summarized in Figure 5.

**FIGURE 5:**
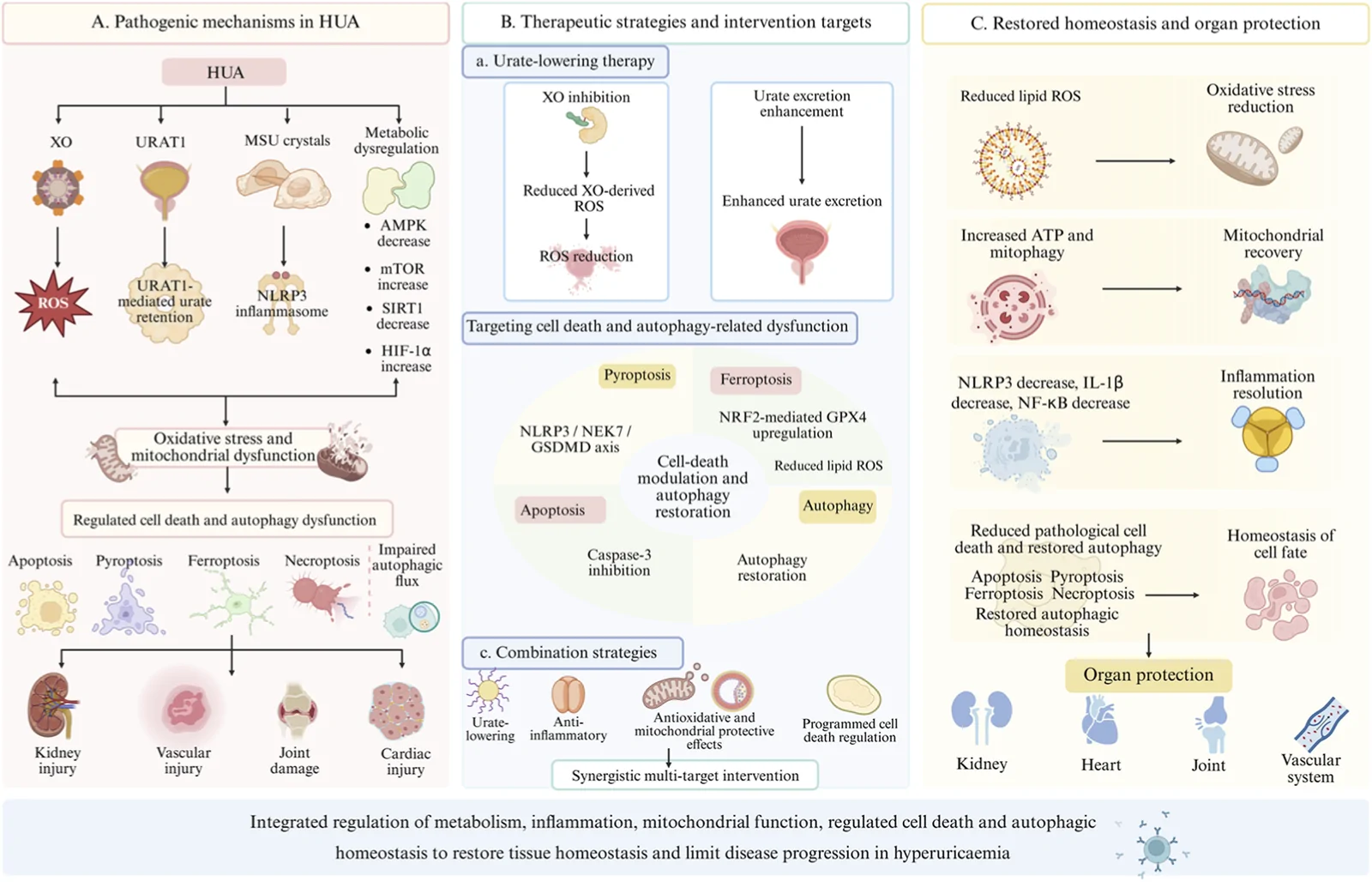
Integrated therapeutic framework targeting urate metabolism and PCD in HUA: **(A)** The HUA-associated pathogenic mechanisms include overproduction and retention of urate, oxidative stress, inflammation, activation of the inflammasome, mitochondrial dysfunction and dysregulated PCD and autophagy. **(B)** The therapeutic strategies are inhibition of urate production; the enhancement of urate excretion; and modulation of pyroptosis, ferroptosis, apoptosis and autophagy-related pathways. **(C)** These interventions are intended to restore redox, inflammatory, metabolic, and cellular homeostasis, thus providing renal and cardiovascular protection as well as benefit to joints and other involved organs.

### 6.2 Targeting PCD

HUA is not only associated with gout but also orchestrates chronic injury in the kidney and cardiovascular system. There is growing evidence that pathways of regulated cell death such as pyroptosis, apoptosis and ferroptosis can at least in part promote disease progression and an autophagy-related dysfunction may modify cellular stress responses and susceptibility to death. Soluble urate and MSU crystals induce cellular injury and inflammation through distinct signalling pathways, thereby contributing to target-organ damage. These findings allow clinicians to consider PCD regulation in the design of novel future therapies, where elucidating additional cellular signalling pathways may be an effective therapeutic goal.

In regard to therapy, in HN the classical anti-ferroptotic role of DIOS is potentially mediated by targeting the BNIP3/NRF2/GPX4 axis to inhibit tubular ferroptosis, resulting in significant renoprotection and allowing consideration as a new therapeutic strategy for nephroprotection (Hu C. et al., 2025). Conversely, the ferroptosis inhibitor Fer-1 diminishes renal urate reabsorption and is linked with decreased key markers of ferroptosis, which provides another target for intervention (Li Y. et al., 2024). These results corroborate the idea that modulating intracellular ROS and lipid peroxidation can protect against urate-induced tissue damage.

Pyroptosis is an ancient and powerful innate immune effector mechanism that plays a key role in HUA and gout, closely associated with NLRP3 inflammasome activation. Dysregulated autophagy may be a mechanism that contributes to NLRP3-dependent pyroptosis, which in turn aggravates renal injury, suggesting that targeting autophagy or inflammasome activation may have therapeutic significance (Hu et al., 2022). Overall, gallic acid transcriptionally activates the NRF2 pathway and inhibits NLRP3 activation and pyroptosis, alleviating gouty inflammation (Lin et al., 2020). Anemoside B4 also suppresses the activation of NLRP3 and pyroptosis by targeting NIMA-related kinase 7 (NEK7) to regulate NF-κB signalling in acute gouty arthritis induced by MSU (Ni et al., 2025). Furthermore, ω-3 polyunsaturated fatty acids alleviate renal pyroptosis via the GPR120/NF-κB axis, inhibiting disease progression in HN (Yang et al., 2024). Interestingly, MSU crystals also induce NLRP3-independent necrotic cell death, which can be inhibited by simultaneous targeting of multiple signalling pathways, thereby opening up alternative therapeutic avenues for the treatment of gout (Zhong et al., 2022).

Similarly, apoptotic pathways are key to urate-associated renal damage. RHF reduces tubular epithelial apoptosis and inflammation by targeting the p53-dependent intrinsic pathway, while also blocking NF-κB signalling (Tang et al., 2023). This highlights the usefulness of intervening pathways involved in apoptosis, since the modulation of Bcl-2/Bax balance and the inhibition of caspase-3 activation are also able to significantly reduce urate-induced apoptosis. Collectively, these results imply that different types of PCD are linked by intricate signalling networks and are often coupled to pro-inflammatory cascades, rendering multi-targeting strategies ideally suited for augmenting therapeutic efficacy in clinical settings.

Targeting ferroptosis, pyroptosis and apoptosis, together with restoration of appropriate autophagic homeostasis, offers an integrative conceptual basis for therapeutics in HUA, gout and associated renal disease. Nonetheless, these findings are primarily based on *in vitro* and preclinical models, and the regulatory interplay between cell death pathways, along with selective and safe targeting of such interventions, remains to be elucidated. Integrated approaches based on small molecules, natural compounds and pathway-directed modulation are beginning to broaden their translational potential.

### 6.3 Combination therapeutic strategies

The evolution of diseases associated with HUA is not a simple consequence of increased urate concentrations but represents an ongoing interaction between aberrant urate metabolism, oxidative stress, chronic inflammatory hyperactivity, dysfunctional mitochondria and excessive PCD. Such persistent HUA results in chronic injury to the kidney, vasculature, joints and myocardium, wherein there is impaired repair, progressive loss of cellular homoeostasis and continuous tissue remodelling. Urate-lowering therapy may decrease production or increase excretion but rarely restores the established pathological milieu to baseline. Combination strategies may also support urate control and improve metabolic parameters in mild HUA (Oshima et al., 2019).

Thus, approaches targeting urate production and excretion have provided unambiguous synergy. Glycine co-supplementation with tryptophan lowers serum urate (Oshima et al., 2019). This study demonstrates that lesinurad plus allopurinol has additive urate-lowering, as well as tissue-protective effects in experimental HUA at the biochemical, molecular and cellular levels (Alghamdi et al., 2020). The paired use of the whey protein-derived peptides PEW and LLW reduces serum urate, creatinine and blood urea nitrogen while increasing fractional urate excretion, suggesting additional potential for dietary intervention (Qi et al., 2023). Taken together, these findings suggest that dysregulation of urate involves multiple transport and synthetic pathways and implies that single-target intervention is unlikely to completely halt the course of the disease; rather, coordinated regulation of production, transport and excretion is more likely to restore metabolic homeostasis.

Combination therapy also has the advantageous feature of being able to simultaneously modulate inflammatory amplification, oxidative stress and PCD networks. In HUA, persistent activation of NF-κB, MAPK and the NLRP3 inflammasome results in the expansion of pyroptosis, apoptosis and necroptosis, while ROS accumulation, ATP depletion and mitochondrial depolarisation further aggravate cellular injury. Allopurinol with Dahuang Mudan decoction has been proven to be effective for early-stage CKD complicated by HUA in a clinical study (Fang et al., 2024). Similarly, parsley along with celery alleviates experimental HUA much more successfully, suggesting synergistic anti-inflammatory, antioxidant and metabolic activities of food-origin compounds (Soliman et al., 2020). Such observations underscore that HUA-related tissue injury arises from common but distinct mechanisms of soluble urate action and immune-mediated damage by MSU crystals. Oxidative, mitochondrial and metabolic stress is mostly driven by soluble urate, while MSU crystal deposition and phagocytosis strongly activate inflammatory signalling; downstream involvement of apoptosis, pyroptosis, ferroptosis and necroptosis depends on tissue type and experimental context.

Thus, therapeutic strategies are moving away from conventional urate lowering to holistic modulation of metabolism, inflammation, oxidative stress and cell fate. It is apparent that critical signalling nodes, including AMPK, SIRT1, NRF2 and mTOR, appear to be optimal targets for restoring mitochondrial function, autophagic balance and energy homeostasis. Yet, PCD also has key physiological roles regulating cellular turnover and tissue renewal; blanket inhibition may frustrate repair. Accordingly, future strategies should be based on a stage-specific and tissue-informed stratification that sets the right level of suppression of pathological cell death against the need to preserve homeostatic function.

## 7 Discussion

HUA was long accepted as a disorder of urate metabolism, yet emerging evidence highlights the role of oxidative stress, inflammation and immune dysregulation. Increasingly, PCD and related stress responses are implicated in HUA-associated organ injury. Autophagy and NLRP3-dependent inflammation induced by HUA are also associated with renal injury based on experimental evidence (Wu et al., 2021). High soluble urate can induce oxidative and mitochondrial stress that supports apoptosis, increasing susceptibility to ferroptotic cell death, whereas MSU crystal deposition specifically activates NLRP3 inflammasome signalling and pyroptosis (He M. et al., 2022). In addition, the oxidative stress caused by high UA at the same time can aggravate vascular cell dysfunction (Dogru et al., 2024). Together, these observations identify a role for PCD regulation as a critical determinant of organ damage in HUA.

An interconnected network of complex regulation governs the various distinct PCD modalities that can together drive pathological progression. A common upstream mechanism is oxidative stress, activating several death pathways at once. However, excessive ROS not only stimulates mitochondrial apoptosis but also induces lipid peroxidation and ferroptosis. Crosstalk between inflammasome activation and autophagy is also observed, in that altered autophagic activity characterizes experimental HN along with increased NLRP3-dependent inflammatory signalling (Wu et al., 2021), suggesting that dysregulated autophagy may modulate inflammasome activity, thus promoting renal inflammation and injury. MSU-induced necrosis involves several signalling pathways, including mechanisms associated with necroptosis (Zhong et al., 2022). In the case of PCD, however, they should be recognized as interconnected pathways as their interplay is likely fundamental for chronic disease progression.

The current state of research has largely focused on the renal, cardiovascular and metabolic complications of HUA. The kidney is the primary organ for urate excretion, and susceptibility to injury directly by urate also impacts fibrotic interstitial responses, with GSDMD-mediated pyroptosis in tubular epithelial cells contributing to this process (Ma et al., 2023). In the cardiovascular system, urate contributes to ECD, inflammation and oxidative stress, contributing to atherosclerosis acceleration and also possibly hypertensive effects (Lee et al., 2021). PCD participates in the pathophysiological process of adipose inflammation and IR, leading to metabolic imbalance in metabolism-related tissues. At present, however, most evidence is from *in vitro* and animal studies with limited clinical validation. However, the temporal dynamics and key regulatory nodes linking different PCD pathways are still insufficiently mapped out and need to be further scrutinized in detail. PCD is essential for tissue homeostasis by regulating highly coordinated cell removal processes.

As progress continues, PCD is coming forward as a new therapeutic target in the treatment of HUA. Strategies that inhibit NLRP3 inflammasome activation or reduce oxidative stress and inflammation have proved effective in preventing urate-mediated tissue damage (Riaz et al., 2023). It remains insufficiently established if commonly used urate-lowering therapies directly modulate PCD independently of urate reduction, and this warrants further investigation. It is therefore crucial for subsequent studies to identify the important regulatory nodes governing PCD networks and assess whether urate-lowering strategies can be complemented by PCD-targeted strategies for therapeutic benefits.

## Glossary

ABCG2: ATP-binding cassette subfamily G member 2
ADAMTS-4: a disintegrin and metalloproteinase with thrombospondin motifs 4
ADAMTS-5: a disintegrin and metalloproteinase with thrombospondin motifs 5
AIF: apoptosis-inducing factor
AIF-1: allograft inflammatory factor 1
AIM2: absent in melanoma 2
AKI: acute kidney injury
AKT: protein kinase B
ALDH2: aldehyde dehydrogenase 2
AMPK: AMP-activated protein kinase
ASC: apoptosis-associated speck-like protein containing a CARD
ATP: adenosine triphosphate
Bax: B-cell lymphoma 2-associated X protein
Bcl-2: B-cell lymphoma 2
BNIP3: BCL2 interacting protein 3
BRD4: bromodomain-containing protein 4
cAMP: cyclic adenosine monophosphate
CHOP: C/EBP homologous protein
CKD: chronic kidney disease
CMPK2: cytidine/uridine monophosphate kinase 2
CPPD: calcium pyrophosphate dihydrate
CPT1B: carnitine palmitoyltransferase 1B
CTRP3: C1q/TNF-related protein 3
DAMP: damage-associated molecular pattern
DDX3X: DEAD-box helicase 3 X-linked
DIOS: diosmetin
DMT1: divalent metal transporter 1
DOX: doxorubicin
DRP1: dynamin-related protein 1
ECD: endothelial dysfunction
eNOS: endothelial nitric oxide synthase
ER: endoplasmic reticulum
ERK1/2: extracellular signal-regulated kinase 1/2
EZH2: enhancer of zeste homolog 2
Fer-1: Ferrostatin-1
FIS1: mitochondrial fission 1 protein
FPS: fucoidan
FTH1: ferritin heavy chain 1
FTL1: ferritin light chain 1
GLUT9: glucose transporter 9
GME: Gnetum montanum extract
GPX4: glutathione peroxidase 4
GSDMD: gasdermin D
GSDME: gasdermin E
GSH: reduced glutathione
HCQ: hydroxychloroquine
HIF-1α: hypoxia-inducible factor 1 alpha
HIF-2α: hypoxia-inducible factor 2 alpha
HN: hyperuricaemic nephropathy
HUA: hyperuricaemia
HUVECs: human umbilical vein endothelial cells
IR: insulin resistance
JAK: Janus kinase
JAK2: Janus kinase 2
LC3: microtubule-associated protein 1 light chain 3
LDH: lactate dehydrogenase
MAPK: mitogen-activated protein kinase
MDM2: MDM2 proto-oncogene
MetS: metabolic syndrome
miR-295: microRNA-295
MLKL: mixed lineage kinase domain-like protein
MSU: monosodium urate
mTOR: mechanistic target of rapamycin
mtROS: mitochondrial reactive oxygen species
MyD88: myeloid differentiation primary response 88
NASH: non-alcoholic steatohepatitis
NCOA4: nuclear receptor coactivator 4
NEK7: NIMA-related kinase 7
NET: neutrophil extracellular trap
NETosis: neutrophil extracellular trap formation
NF-κB: nuclear factor kappa B
N-GSDMD: N-terminal fragment of gasdermin D
NKA: Na^+^-K^+^-ATPase
NLRP3: NOD-like receptor family pyrin domain containing 3
NO: nitric oxide
NOX1: NADPH oxidase 1
NRF2: nuclear factor erythroid 2–related factor 2
OAT1: organic anion transporter 1
OAT3: organic anion transporter 3
P2X7: P2X purinoceptor 7
P2Y14R: P2Y14 receptor
Panx-1: pannexin 1
PCD: programmed cell death
PI3K: phosphoinositide 3-kinase
PINK1: PTEN-induced kinase 1
PISD: phosphatidylserine decarboxylase
p-JUN: phosphorylated JUN
PPARγ: peroxisome proliferator-activated receptor gamma
PRDX2: peroxiredoxin 2
PTECs: proximal tubular epithelial cells
RHF: renal herb formula
RIG-I: retinoic acid-inducible gene I
RIPK1: receptor-interacting serine/threonine-protein kinase 1
RIPK3: receptor-interacting serine/threonine-protein kinase 3
ROS: reactive oxygen species
SIRT1: sirtuin 1
SLC22A12: solute carrier family 22 member 12
SLC2A9: solute carrier family 2 member 9
SLC7A11: solute carrier family 7 member 11
SOCS1: suppressor of cytokine signalling 1
STAT3: signal transducer and activator of transcription 3
TBHQ: tert-butylhydroquinone
TEA: total extract of Abelmoschus manihot L. flower
TGF-β1: transforming growth factor beta 1
TLR4: Toll-like receptor 4
TNF-α: tumour necrosis factor-α
TUNEL: terminal deoxynucleotidyl transferase dUTP nick end labeling
TXNIP: thioredoxin-interacting protein
UA: uric acid
UNx: uninephrectomy
URAT1: urate transporter 1
XO: xanthine oxidase
XOR: xanthine oxidoreductase
